# Ultrasound-Assisted Aqueous Extraction of *Origanum sipyleum* and *Sideritis pisidica*: LC–MS/MS Phenolic Profiles and Bioactivities

**DOI:** 10.3390/molecules31213956

**Published:** 2026-11-02

**Authors:** Ümmühan Ünlü

**Affiliations:** Department of Health Care Services, Atatürk Vocational School of Health Services, Afyonkarahisar Health Sciences University, Afyonkarahisar 03030, Türkiye; ummuhanunluafsu@gmail.com

**Keywords:** *Origanum sipyleum*, *Sideritis pisidica*, phenolic compounds, LC–MS/MS, enzyme inhibition

## Abstract

*Origanum sipyleum* and *Sideritis pisidica* are endemic Turkish Lamiaceae species with previously reported phytochemistry and bioactivities, but matched-condition comparisons remain limited. This study aimed to compare aqueous extracts prepared by ultrasound-assisted extraction (5 g/100 mL water, 40 kHz, 360 W nominal ultrasonic power, 3 h, approximately 40 °C). A previously established 28-compound panel was analyzed by LC–MS/MS, alongside total phenolic and flavonoid contents, six antioxidant assays, and five enzyme-inhibitory assays. Eighteen and 19 phenolic compounds were detected in *O. sipyleum* and *S. pisidica*, respectively. Protocatechuic acid predominated in *O. sipyleum* (2334.15 µg/g extract), whereas chlorogenic acid predominated in *S. pisidica* (3525.31 µg/g extract). *O. sipyleum* exhibited significantly higher total phenolic and flavonoid contents and greater radical-scavenging and reducing activities, while phosphomolybdenum and ferrous-ion chelating activities were statistically comparable. Butyrylcholinesterase and α-amylase inhibitor-equivalent activities were statistically comparable between the species, whereas *S. pisidica* exhibited higher acetylcholinesterase-inhibitory (3.14 versus 2.94 mg galantamine equivalents/g extract) and tyrosinase-inhibitory activities (23.90 versus 21.01 mg kojic acid equivalents/g extract). *O. sipyleum* also displayed α-glucosidase inhibition (319.56 mg acarbose equivalents/g extract), while no inhibition was detected for *S. pisidica* within 0.1–5.0 mg/mL. This comparison identifies contrasting targeted phenolic profiles and assay-dependent in vitro responses without establishing compound-level causality.

## 1. Introduction

The Lamiaceae family comprises a diverse group of aromatic and medicinal plants that have long been used in traditional practices throughout the Mediterranean region and Anatolia. Among its members, species belonging to the genera *Origanum* L. and *Sideritis* L. are widely consumed in Türkiye as herbal or “mountain” teas, typically prepared as hot-water infusions, and are valued for their characteristic aroma and traditional uses [1,2,3]. Türkiye represents an important center of diversity for both genera and contains numerous endemic taxa. Their phytochemical composition, particularly their phenolic constituents, has attracted considerable attention because phenolic compounds exhibit diverse redox properties and, in some experimental systems, enzyme-inhibitory activities. However, the contribution of individual phenolic compounds to the measured activities of a particular crude plant extract cannot be assumed solely from their detection or concentration.

*Origanum sipyleum* L. is an endemic Turkish species distributed mainly in western Anatolia, particularly around Mount Sipylus (Spil Dağı, Manisa Province) [4,5]. Its aerial parts have traditionally been consumed as herbal tea and used as a culinary flavoring [5,6]. Earlier investigations focused primarily on its essential-oil composition and subsequently on its phenolic constituents, antioxidant activity, and other biological properties using different extraction systems [4,5,6,7]. Importantly, Zengin et al. [3] investigated ethyl acetate, methanol, and water extracts of *O. sipyleum* and demonstrated distinct phytochemical, antioxidant, and enzyme-inhibitory profiles among the extracts; the aqueous extract exhibited particularly notable antioxidant and acetylcholinesterase-inhibitory activities. Thus, the biological and phytochemical properties of *O. sipyleum*, including its aqueous extract, have been investigated previously. Nevertheless, quantitative characterization using a targeted LC–MS/MS phenolic panel combined with a broad set of antioxidant and enzyme-inhibitory assays under standardized ultrasound-assisted aqueous extraction conditions remains relevant for a more detailed comparison with other traditionally consumed Lamiaceae species.

*Sideritis pisidica* Boiss. et Heldr. is another endemic Turkish Lamiaceae species belonging to a genus whose members are widely consumed as mountain tea. However, genus-level ethnobotanical evidence should not be interpreted as confirmation of documented traditional use specifically for *S. pisidica*. Previous research demonstrates that this species cannot be regarded as phytochemically unexplored. Ozkan [8] compared the phenolic composition of ethanolic extracts and aqueous infusions of several *Sideritis* species, including *S. pisidica*, and identified phenolic constituents by HPLC, thereby providing early evidence on the composition of its water-based preparations. Subsequently, Deveci et al. [9] investigated the chemical composition, antioxidant, anticholinesterase, and anti-urease activities of the essential oil and hexane, acetone, and methanol extracts of *S. pisidica*. The methanol extract displayed the strongest antioxidant performance in most of the assays investigated, whereas enzyme-inhibitory effects varied according to the extraction system. More recently, Erdoğmuş et al. [10] characterized the phytochemical profile of *S. pisidica* and evaluated its biological effects using in vitro melanoma-cell and in silico approaches. Rosmarinic acid was reported as a prominent constituent, together with caffeic acid and several flavonoid derivatives, including luteolin 7-glucoside, hyperoside, hesperidin, apigenin 7-glucoside, kaempferol, and luteolin. Collectively, these studies establish previous knowledge of the chemistry and biological properties of *S. pisidica*; however, differences in extraction procedures, analytical platforms, and bioactivity assays limit direct comparison across studies.

Previous comparative research has also included related species from these genera. Nakiboglu et al. [11] investigated the antioxidant capacities of *O. sipyleum* and *Sideritis sipylea* using water, ethanol, methanol, and acetone extracts, demonstrating that antioxidant responses varied according to the species, extraction solvent, and assay employed. Importantly, *S. sipylea* is distinct from *S. pisidica*, the species examined in the present study. Moreover, the earlier investigation focused on total phenolic content and selected antioxidant endpoints rather than the combined targeted quantification of 28 phenolic compounds and evaluation of antioxidant and enzyme-inhibitory activities applied here. Thus, although comparative studies involving these genera already exist, the specific analytical and biological comparison undertaken in the present investigation remains insufficiently characterized.

The selection of *O. sipyleum* and *S. pisidica* for direct comparison was based on their shared membership in the Lamiaceae family, their endemic status in Türkiye, and the availability of previous phytochemical and bioactivity investigations that provide species-specific scientific context [3,8,9]. Although both species have been investigated individually, existing evidence differs considerably in extraction conditions, analytical coverage, and biological endpoints. Importantly, the comparative rationale extends beyond methodological standardization: evaluating these two species under matched conditions provides an opportunity to determine whether differences in targeted phenolic composition and total phenolic responses are accompanied by consistent differences in antioxidant and enzyme-inhibitory activities, or whether the relative biological responses vary according to the assay and enzyme target. This question cannot be resolved reliably by comparing results obtained independently using different extraction and analytical procedures.

Extraction conditions are among the major factors influencing the qualitative and quantitative composition of plant extracts and, consequently, their measured in vitro bioactivities. Water is particularly relevant for species traditionally consumed as herbal teas because it represents the solvent used in conventional infusions. Ultrasound-assisted extraction (UAE) has emerged as an efficient extraction approach that promotes solvent penetration and mass transfer through acoustic cavitation and can improve the recovery of phenolic compounds while reducing extraction time compared with many conventional procedures [12,13]. Accordingly, the combination of UAE with water provides a solvent-conscious approach suitable for the controlled extraction of relatively polar constituents, including several phenolic acids and flavonoid glycosides. In the present study, water was selected because of its relevance to aqueous herbal preparations, while UAE was employed to establish a common laboratory extraction procedure for the two species rather than to reproduce conventional tea preparation. The comparability of the resulting extracts depends on the consistent control of extraction parameters and does not, by itself, demonstrate the reproducibility or optimality of the procedure. Furthermore, prolonged sonication, temperature fluctuations, and cavitation-associated chemical changes may affect the stability of certain phenolic constituents [14]. Nevertheless, UAE performed under controlled laboratory conditions should not be considered equivalent to household infusion, because sonication, extraction time, temperature, and mass-transfer conditions differ substantially from traditional preparation.

Phenolic constituents of Lamiaceae species are also widely investigated for their in vitro antioxidant and enzyme-inhibitory properties. Because antioxidant activity involves multiple chemical mechanisms, the use of complementary assays based on radical scavenging, reducing capacity, total antioxidant capacity, and metal chelation can provide a broader characterization than any single assay alone. Similarly, acetylcholinesterase (AChE), butyrylcholinesterase (BChE), tyrosinase, α-amylase, and α-glucosidase represent frequently investigated enzyme targets in the biological screening of plant extracts [15,16]. These enzymes were selected to assess distinct biochemical targets rather than to investigate a single therapeutic mechanism. AChE and BChE are cholinesterases involved in the hydrolysis of acetylcholine, although their physiological roles and substrate specificities differ [17]. Tyrosinase catalyzes key reactions in melanin biosynthesis [18], whereas α-amylase and α-glucosidase are involved in carbohydrate digestion [19]. Their inclusion therefore enables a broader in vitro screening of enzyme-inhibitory responses across functionally different targets [15,16]. Nevertheless, inhibition measured in cell-free assays does not establish physiological or clinical efficacy. Furthermore, crude extracts comprise complex mixtures of phenolic and non-phenolic constituents, and associations between individual compounds and measured biological activities should therefore be interpreted cautiously unless supported by isolation, activity-guided fractionation, or mechanistic studies.

Against this background, the present study was designed to directly compare ultrasound-assisted aqueous extracts of *O. sipyleum* and *S. pisidica* using identical extraction, analytical, and bioactivity-assay conditions. Although previous studies have investigated the phytochemical composition and biological activities of these species separately [3,8,9], comparative research involving other *Origanum* and *Sideritis* species has also been reported [11]. However, these investigations differ in their selected species, extraction procedures, analytical coverage, and biological endpoints. In particular, previous research has not established a matched-condition comparison of the targeted phenolic profiles of *O. sipyleum* and *S. pisidica* alongside their responses across the same complementary antioxidant and enzyme-inhibitory assays. To address this gap, the present study applied a common ultrasound-assisted water extraction protocol, targeted LC–MS/MS quantification of 28 phenolic compounds, six complementary antioxidant assays, and five enzyme-inhibitory assays to both species. The central research question was whether these species exhibit distinct targeted phenolic signatures and assay-dependent bioactivity patterns when evaluated under identical experimental conditions. Accordingly, the objectives were to (i) characterize and quantify their phenolic constituents using a targeted LC–MS/MS panel; (ii) determine their total phenolic and flavonoid contents; (iii) evaluate antioxidant capacity using six complementary assays; and (iv) assess inhibitory activities against AChE, BChE, tyrosinase, α-amylase, and α-glucosidase. Beyond generating a matched analytical dataset, the scientific contribution of this study is to establish whether two endemic Lamiaceae species with previously documented but differently characterized phytochemical and biological properties exhibit consistent or target-dependent differences in their aqueous-extract bioactivities. Specifically, the comparison addresses whether higher total phenolic and flavonoid responses and differences in predominant targeted phenolic constituents are accompanied by uniformly higher antioxidant and enzyme-inhibitory activities, or whether distinct biochemical assays reveal divergent response patterns. By examining these relationships under identical experimental conditions, the study provides new comparative evidence concerning the extent to which phytochemical differences are accompanied by assay-dependent biological responses. These observations are interpreted at the crude-extract level and do not establish causal effects of individual phenolic constituents.

## 2. Results

### 2.1. Extraction Yield and Targeted LC–MS/MS Phenolic Profiles

Three independent aqueous extracts were prepared for each species (*n* = 3). The mean extraction yield was 40.04 ± 2.14% for *O. sipyleum* and 16.82 ± 1.41% for *S. pisidica*, corresponding to an approximately 2.4-fold higher yield for *O. sipyleum*. The values represent the mean ± SD of three independently prepared extracts. All subsequent phytochemical and bioactivity analyses were performed independently using these three extracts.

Targeted LC–MS/MS analysis was performed for 28 phenolic compounds. Of these, 18 compounds were detected in *O. sipyleum* and 19 in *S. pisidica* (Table 1).

**Figure 1:**
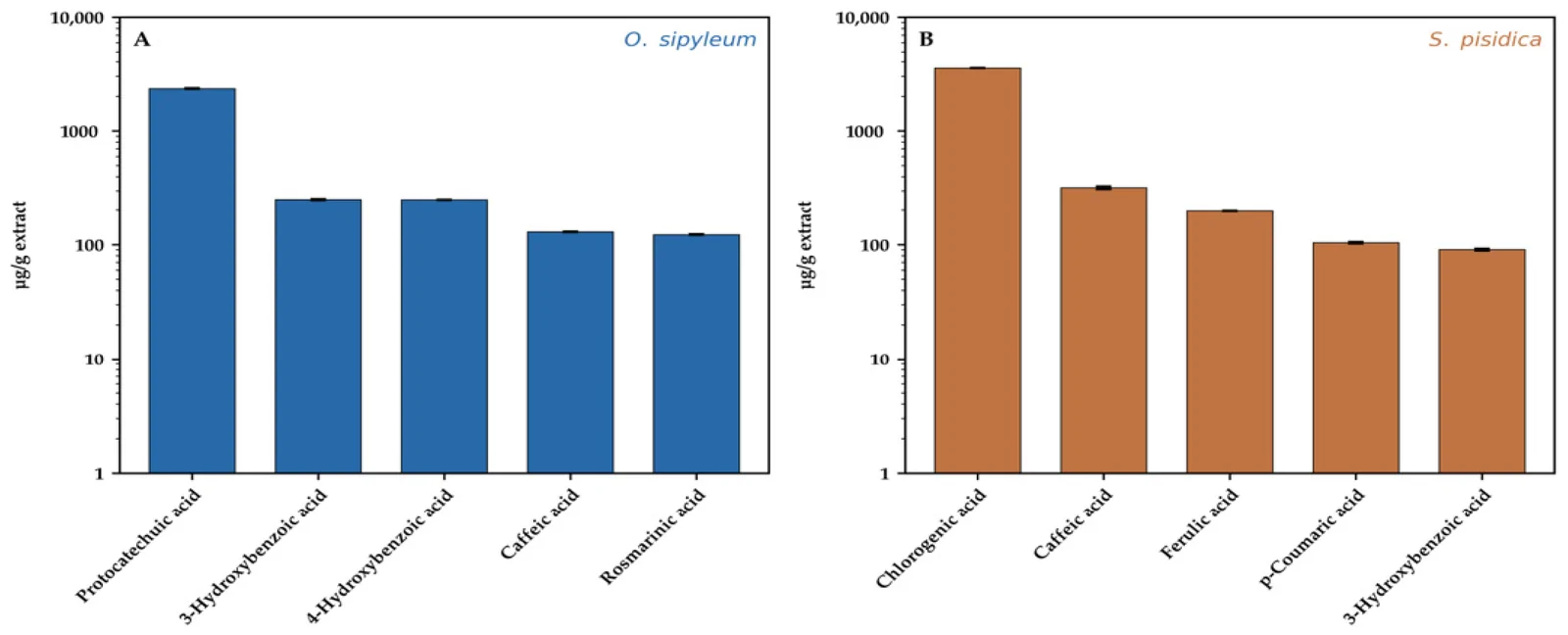
Comparative concentrations of the five predominant phenolic compounds identified by LC–ESI–MS/MS in the ultrasound-assisted aqueous extracts of (**A**) *Origanum sipyleum* and (**B**) *Sideritis pisidica*. Data are expressed as mean ± standard deviation (SD) of three independently prepared extracts (*n* = 3), in µg/g extract. Error bars represent SD. The y-axes are presented on a logarithmic scale to facilitate visualization of differences in compound concentrations.

The compounds 3,4-dihydroxyphenylacetic acid, (−)-epicatechin, taxifolin, sinapic acid, 2-hydroxycinnamic acid, eriodictyol, pinoresinol, and quercetin were not detected in either species. Gallic acid and hesperidin were detected only in *S. pisidica*, whereas kaempferol was detected only in *O. sipyleum*. Overall, the two species exhibited clearly distinct quantitative phenolic profiles. The contrasting distributions of the five predominant phenolic compounds in each extract are illustrated in Figure 1, highlighting the predominance of protocatechuic acid in *O. sipyleum* and chlorogenic acid in *S. pisidica*.

In *O. sipyleum*, protocatechuic acid was the predominant quantified phenolic compound, reaching 2334.15 ± 24.20 µg/g extract. It was followed by 3-hydroxybenzoic acid (252.54 ± 3.97 µg/g), 4-hydroxybenzoic acid (251.45 ± 1.48 µg/g), caffeic acid (129.31 ± 0.89 µg/g), and rosmarinic acid (122.23 ± 1.10 µg/g). Among the quantified flavonoids, apigenin (46.99 ± 0.65 µg/g), luteolin (39.37 ± 0.78 µg/g), and kaempferol (35.09 ± 2.86 µg/g) were present at appreciable concentrations. In contrast, chlorogenic acid (4.06 ± 0.14 µg/g), ferulic acid (2.30 ± 0.11 µg/g), verbascoside (0.34 ± 0.03 µg/g), luteolin 7-glucoside (0.18 ± 0.01 µg/g), and hyperoside (0.44 ± 0.02 µg/g) were detected at relatively low levels. Gallic acid and hesperidin were not detected in *O. sipyleum*.

The phenolic profile of *S. pisidica* was dominated by chlorogenic acid, which was quantified at 3525.31 ± 15.77 µg/g extract. The next most abundant compounds were caffeic acid (320.19 ± 10.05 µg/g), ferulic acid (196.75 ± 1.20 µg/g), *p*-coumaric acid (103.88 ± 1.88 µg/g), 4-hydroxybenzoic acid (90.92 ± 1.26 µg/g), and 3-hydroxybenzoic acid (90.43 ± 2.08 µg/g). Protocatechuic acid was present at 53.38 ± 1.47 µg/g, while syringic acid and verbascoside were quantified at 48.50 ± 1.27 and 33.37 ± 0.73 µg/g, respectively. Gallic acid (28.04 ± 0.75 µg/g) and hesperidin (1.14 ± 0.07 µg/g) were detected exclusively in *S. pisidica*. Among the quantified flavonoids, apigenin (9.28 ± 0.50 µg/g), luteolin (3.71 ± 0.21 µg/g), luteolin 7-glucoside (2.30 ± 0.10 µg/g), apigenin 7-glucoside (8.37 ± 0.40 µg/g), and hyperoside (0.73 ± 0.01 µg/g) were detected, whereas kaempferol was not detected. Among the phenolic compounds detected in both species, *O. sipyleum* contained significantly higher concentrations of protocatechuic acid, 3-hydroxybenzoic acid, 4-hydroxybenzoic acid, rosmarinic acid, luteolin, and apigenin. In contrast, *S. pisidica* contained significantly higher concentrations of (+)-catechin, chlorogenic acid, caffeic acid, syringic acid, *p*-coumaric acid, ferulic acid, verbascoside, luteolin 7-glucoside, hyperoside, and apigenin 7-glucoside (*p* < 0.05). These findings indicate distinct phenolic signatures for the two ultrasound-assisted aqueous extracts. When normalized to the initial dry plant material, protocatechuic acid remained the predominant quantified phenolic compound in O. sipyleum (934.59 µg/g dry plant), whereas chlorogenic acid remained predominant in S. pisidica (592.96 µg/g dry plant). Notably, the caffeic acid contents, which differed significantly on an extract basis, were numerically comparable after normalization to dry plant material (51.78 and 53.86 µg/g dry plant for *O. sipyleum* and *S. pisidica*, respectively; Table 1). These findings highlight the importance of extraction yield when comparing the quantities of recovered phenolic constituents between species.

### 2.2. Total Phenolic and Flavonoid Contents

The total phenolic content (TPC) and total flavonoid content (TFC) of the aqueous extracts are presented in Table 2. *O. sipyleum* exhibited a significantly higher TPC (118.88 ± 4.07 mg GAE/g extract) than *S. pisidica* (47.88 ± 0.53 mg GAE/g extract), corresponding to an approximately 2.5-fold difference (*p* < 0.05). A similar pattern was observed for TFC, with *O. sipyleum* yielding 32.67 ± 0.74 mg RE/g extract compared with 13.81 ± 0.28 mg RE/g extract for *S. pisidica*, representing an approximately 2.4-fold difference (*p* < 0.05). Thus, *O. sipyleum* exhibited significantly higher TPC and TFC values under the extraction conditions employed. After normalization to the initial dry plant material, *O. sipyleum* also exhibited higher TPC (47.60 versus 8.05 mg GAE/g dry plant) and TFC (13.08 versus 2.32 mg RE/g dry plant) than *S. pisidica*, respectively (Table 2).

### 2.3. Antioxidant Activities

The antioxidant activities of the ultrasound-assisted aqueous extracts were evaluated using six complementary assays (Table 3). These assays reflect distinct chemical principles, including radical scavenging (DPPH and ABTS), electron-transfer-based reducing capacity (CUPRAC and FRAP), phosphomolybdenum-based total antioxidant capacity, and ferrous-ion chelation. Accordingly, comparisons were made between the two species within each assay rather than directly comparing the numerical values obtained from different assays. *O. sipyleum* exhibited significantly higher DPPH, ABTS, CUPRAC, and FRAP activities than *S. pisidica* (*p* < 0.05). In the DPPH assay, *O. sipyleum* showed an activity of 286.62 ± 18.36 mg TE/g extract, whereas *S. pisidica* exhibited 90.53 ± 1.31 mg TE/g extract. Similarly, ABTS activity was 403.07 ± 19.88 mg TE/g extract for *O. sipyleum* and 154.24 ± 12.84 mg TE/g extract for *S. pisidica*.

The same pattern was observed in the reducing-power assays. *O. sipyleum* exhibited CUPRAC and FRAP values of 499.44 ± 22.00 and 463.96 ± 10.53 mg TE/g extract, respectively, while the corresponding values for *S. pisidica* were 189.76 ± 1.95 and 143.11 ± 4.73 mg TE/g extract. These values corresponded to approximately 2.6-fold higher CUPRAC and 3.2-fold higher FRAP activities in *O. sipyleum*.

**Figure 2:**
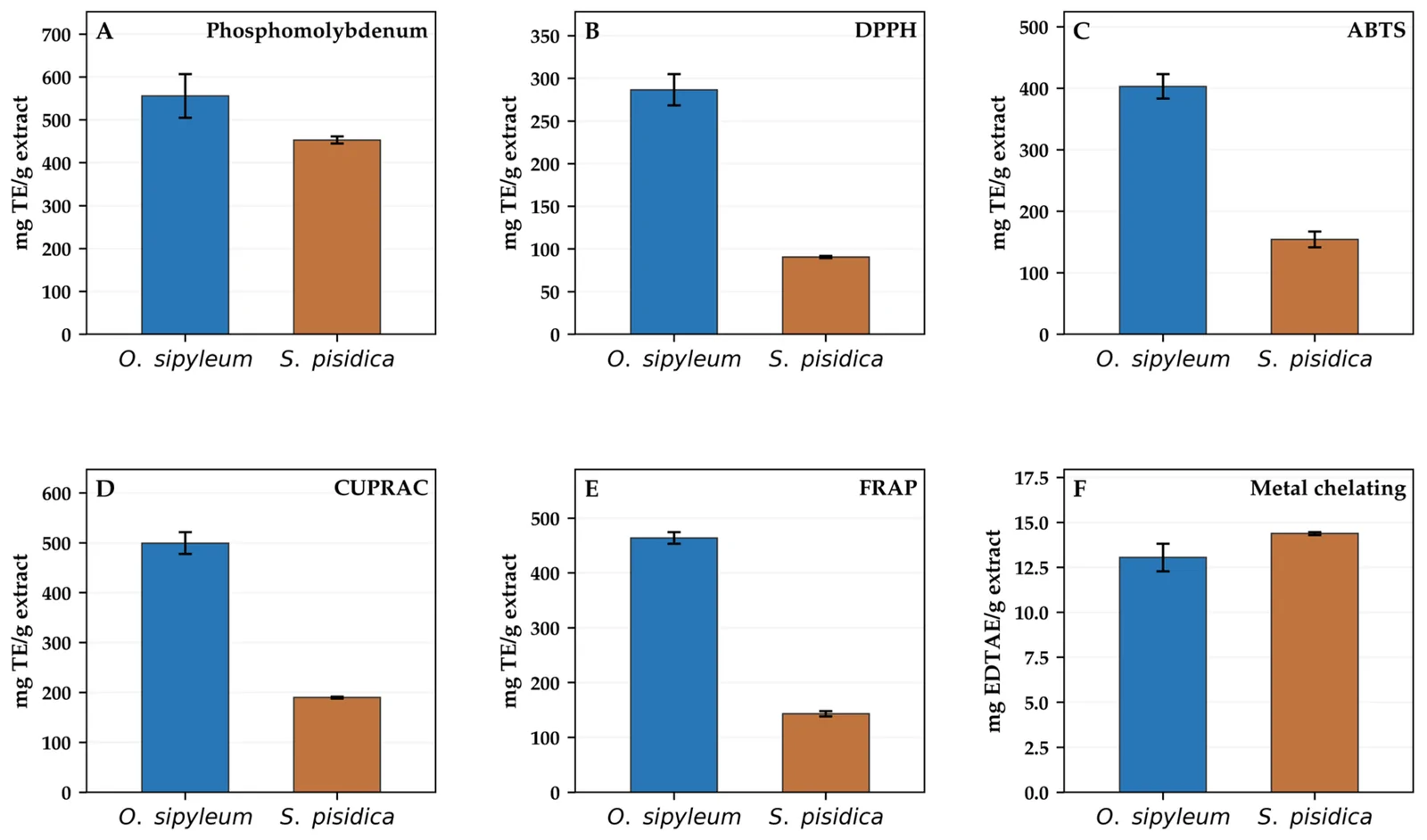
Comparative antioxidant activities of the ultrasound-assisted aqueous extracts of *Origanum sipyleum* and *Sideritis pisidica* determined using six complementary assays: (**A**) phosphomolybdenum, (**B**) DPPH, (**C**) ABTS, (**D**) CUPRAC, (**E**) FRAP, and (**F**) ferrous-ion chelating activity. Results are expressed as mean ± standard deviation (SD) of three independently prepared extracts (*n* = 3). Antioxidant activities are reported as mg Trolox equivalents (TE)/g extract for assays (**A**–**E**) and mg EDTA equivalents (EDTAE)/g extract for assay (**F**). Error bars represent SD. Each assay is presented separately because the measurements reflect distinct chemical mechanisms and are not directly comparable across assays.

In contrast, no statistically significant difference was observed between the two species in the phosphomolybdenum assay. The values were 555.86 ± 50.90 mg TE/g extract for *O. sipyleum* and 453.31 ± 8.37 mg TE/g extract for *S. pisidica* (*p* > 0.05). Ferrous-ion chelating activity was also statistically comparable between the species, with values of 13.05 ± 0.77 and 14.38 ± 0.08 mg EDTAE/g extract for *O. sipyleum* and *S. pisidica*, respectively (*p* > 0.05). The comparative antioxidant activity profiles of the two aqueous extracts across all six assays are illustrated in Figure 2, highlighting the assay-dependent differences between the species.

Normalization to the initial dry plant material revealed numerically higher antioxidant activities for *O. sipyleum* across all six assays (Table 3). In particular, phosphomolybdenum and ferrous-ion chelating activities, which did not differ significantly between the extracts on a per-gram-extract basis, showed more pronounced numerical differences when expressed per gram of dry plant material (222.57 versus 76.25 mg TE/g dry plant and 5.23 versus 2.42 mg EDTAE/g dry plant, respectively). These results illustrate the influence of extraction yield on the interpretation of antioxidant capacity expressed relative to the starting plant material.

### 2.4. Enzyme-Inhibitory Activities

The enzyme-inhibitory activities of the ultrasound-assisted aqueous extracts are presented in Table 4. A statistically significant difference was observed between *O. sipyleum* and *S. pisidica* for AChE inhibitor-equivalent activity (*p* < 0.05), whereas BChE inhibition did not differ significantly between the species (*p* > 0.05). AChE-inhibitory activities were 2.94 ± 0.09 and 3.14 ± 0.08 mg GALAE/g extract for *O. sipyleum* and *S. pisidica*, respectively. Similarly, BChE-inhibitory activities were 5.15 ± 0.19 mg GALAE/g extract for *O. sipyleum* and 5.08 ± 0.06 mg GALAE/g extract for *S. pisidica*. A significant difference was observed in tyrosinase inhibition. *S. pisidica* exhibited significantly higher tyrosinase-inhibitory activity (23.90 ± 0.26 mg KAE/g extract) than *O. sipyleum* (21.01 ± 0.54 mg KAE/g extract) (*p* < 0.05). The α-amylase-inhibitory activities of the two extracts were comparable, with values of 34.91 ± 1.11 mg ACE/g extract for *O. sipyleum* and 36.02 ± 1.07 mg ACE/g extract for *S. pisidica*, with no statistically significant difference between the species (*p* > 0.05). A marked difference was observed for α-glucosidase inhibition. *O. sipyleum* exhibited an α-glucosidase-inhibitory activity of 319.56 ± 15.01 mg ACE/g extract, whereas no inhibitory activity was detected for *S. pisidica* under the assay conditions. Therefore, no statistical comparison was performed between the two species for this assay.

The IC_50_ values obtained from concentration–response analyses further characterized the enzyme-inhibitory activities of the two extracts (Table 4).

For AChE inhibition, the IC_50_ values were 1.11 ± 0.01 mg/mL for *O. sipyleum* and 1.05 ± 0.03 mg/mL for *S. pisidica*, while the corresponding BChE values were 1.03 ± 0.01 and 1.04 ± 0.02 mg/mL, respectively. No statistically significant differences were observed between the two extracts in AChE or BChE IC_50_ values (*p* > 0.05). In contrast, *S. pisidica* exhibited a significantly lower tyrosinase IC_50_ value (3.39 ± 0.04 mg/mL) than *O. sipyleum* (3.86 ± 0.10 mg/mL; *p* < 0.05), indicating greater inhibitory potency against this enzyme. For α-amylase, neither extract achieved 50% inhibition at the highest tested concentration (20 mg/mL), and the corresponding IC_50_ values were therefore reported as >20 mg/mL. Regarding α-glucosidase inhibition, an IC_50_ value of 1.75 ± 0.08 mg/mL was obtained for *O. sipyleum*, whereas no IC_50_ value could be determined for *S. pisidica* because inhibition was not detected under the assay conditions. Specifically, no detectable α-glucosidase inhibition was observed for *S. pisidica* at any concentration within the tested range (0.1–5.0 mg/mL). The reference inhibitors consistently exhibited lower IC_50_ values than the plant extracts wherever quantitative comparisons were possible.

## 3. Discussion

### 3.1. Extraction Yield and Comparative Phenolic Profiles

The two species showed a marked difference in extraction yield under identical UAE–water conditions. The mean extraction yield of *O. sipyleum* (40.04 ± 2.14%) was approximately 2.4-fold higher than that of *S. pisidica* (16.82 ± 1.41%). Because the same extraction procedure was applied to both species, this difference indicates a greater recovery of water-extractable material from *O. sipyleum* under the conditions employed. However, extraction yield represents the recovery of the entire soluble fraction and should not be interpreted as a direct measure of phenolic content. Previous studies also demonstrate the strong influence of solvent and extraction procedure on extract recovery. Ozkan et al. [5] obtained a 14.13 ± 3.03% yield from a methanolic *O. sipyleum* extract. Direct comparison with the present yields is nevertheless limited by differences in extraction technique, solvent composition, plant material, and reporting basis.

Targeted LC–MS/MS analysis further demonstrated pronounced differences between the two species. In *O. sipyleum*, protocatechuic acid was the predominant quantified phenolic compound (2334.15 µg/g extract), followed by 3-hydroxybenzoic acid, 4-hydroxybenzoic acid, caffeic acid, and rosmarinic acid. This profile differs substantially from earlier reports on *O. sipyleum*. Ozkan et al. [5], using an ultrasound-assisted methanolic extraction followed by RP-HPLC, reported vitexin and rosmarinic acid as prominent constituents, with rosmarinic acid reaching 870.22 mg/100 g herb. Kaska [6] likewise characterized an ethanolic extract of *O. sipyleum* but reported caffeic acid, epicatechin, and 2,5-dihydroxybenzoic acid among the major phenolics. Zengin et al. [3] subsequently demonstrated that the phytochemical profile of *O. sipyleum* varied considerably among ethyl acetate, methanol, and water extracts, with rosmarinic acid and related phenolics contributing substantially to the LC–MS profile. The predominance of protocatechuic acid observed in the present study therefore appears to represent a distinctive feature of the current UAE–water extract rather than a universal chemotaxonomic characteristic of *O. sipyleum*.

The strong influence of species, organ, and extraction conditions is further illustrated by recent data for the Turkish endemic *O. bilgeri*. Using UAE with ethanol and the same targeted panel of 28 phenolic compounds, Karakus and Sarikurkcu [21] found rosmarinic acid to be the predominant compound in all four investigated organs, reaching 2776, 3236, 3093, and 1500 µg/g extract in the flower, leaf, stem, and root, respectively. In contrast, protocatechuic acid concentrations were substantially lower, ranging from 22.5 to 198 µg/g extract. The root displayed a markedly different profile from the aerial organs because of its high chlorogenic acid (1461 µg/g) and verbascoside (2775 µg/g) contents. These observations reinforce the need to interpret phenolic profiles in relation to taxon, plant organ, extraction solvent, and analytical conditions rather than assuming a uniform profile within the genus *Origanum*.

A different phenolic pattern was observed in *S. pisidica*. Chlorogenic acid was the predominant targeted compound (3525.31 µg/g extract), followed by caffeic acid (320.19 µg/g), ferulic acid (196.75 µg/g), *p*-coumaric acid (103.88 µg/g), and the two hydroxybenzoic acid isomers. Verbascoside was also detected at 33.37 µg/g extract, whereas rosmarinic acid occurred at a comparatively low concentration of 8.60 µg/g. Previous reports on *S. pisidica* show substantial methodological variation. In an aqueous infusion, Ozkan [8] reported naringin (18.63 mg/100 g dried herb) and vitexin (12.33 mg/100 g dried herb) among the more abundant compounds, while rosmarinic acid was not detected. Notably, chlorogenic acid was not included among the reported analytes in that study, whereas several compounds quantified by Özkan, including naringin and vitexin, were not included in the targeted panel used here. Thus, apparent differences between the two studies partly reflect differences in analytical coverage as well as extraction procedure. Deveci et al. [9] further demonstrated solvent-dependent variation in *S. pisidica*. Among hexane, acetone, and methanol extracts, the acetone extract displayed the highest total phenolic content, whereas the methanol extract showed the highest total flavonoid content. However, that investigation did not provide an LC–MS/MS profile of individual non-volatile phenolics comparable to the present dataset. More recently, Erdoğmuş et al. [10] reported rosmarinic acid as the predominant phytochemical in *S. pisidica*, together with caffeic acid, luteolin 7-glucoside, hyperoside, hesperidin, apigenin 7-glucoside, kaempferol, and luteolin. The contrast between the rosmarinic-acid-rich profile reported in that study and the chlorogenic-acid-dominant profile observed here further emphasizes the sensitivity of phytochemical profiles to extraction conditions, sample origin, and analytical methodology.

Comparisons with previously published studies should be interpreted cautiously because differences in plant material, extraction solvents and procedures, analytical techniques, target-analyte coverage, and reporting units limit direct quantitative comparability. Accordingly, the literature findings discussed above are used to contextualize the present results rather than to establish direct numerical rankings across studies.

The phenolic profiles observed in the present study differed substantially from those reported in previous investigations of *Origanum* and *Sideritis* extracts. Under the standardized UAE–water conditions used here, *O. sipyleum* was characterized predominantly by protocatechuic acid and hydroxybenzoic acid derivatives, whereas *S. pisidica* showed a chlorogenic-acid-dominant profile accompanied by other hydroxycinnamic acids. This side-by-side comparison therefore demonstrates clearly differentiated phenolic signatures under identical experimental conditions. However, because the analytical method was restricted to 28 predefined phenolic compounds, the present data should be interpreted as targeted phenolic profiles rather than comprehensive metabolomic fingerprints.

### 3.2. Phenolic Composition and Antioxidant Activities

The differences in the targeted phenolic profiles of the two species were accompanied by marked differences in their total phenolic and flavonoid contents and in several antioxidant assays. *O. sipyleum* exhibited approximately 2.5-fold higher TPC and 2.4-fold higher TFC than *S. pisidica*. This pattern was paralleled by significantly higher DPPH, ABTS, CUPRAC, and FRAP values for *O. sipyleum*. The magnitude of the difference ranged from approximately 2.6-fold for ABTS and CUPRAC to approximately 3.2-fold for DPPH and FRAP. Because these assays probe related but distinct aspects of antioxidant behavior, their broadly consistent response suggests that the *O. sipyleum* aqueous extract possessed greater radical-scavenging and reducing capacity under the experimental conditions used. However, the present data demonstrate an association rather than a formal statistical correlation between phenolic content and antioxidant capacity.

The additional expression of phenolic contents and antioxidant activities relative to the initial dry plant material provides a complementary perspective on the differences between the two species. Importantly, normalization for extraction yield reduced the apparent difference in caffeic acid content between the species, while the differences in several antioxidant parameters became more pronounced. These observations demonstrate that extract-based and dry-plant-based results provide distinct but complementary information: the former characterizes the composition and activity of the recovered extracts, whereas the latter accounts for the amount of extract recovered from the starting plant material. Therefore, both reporting approaches should be considered when interpreting comparative phytochemical and antioxidant data.

Previous studies also support the antioxidant potential of *O. sipyleum*, although direct numerical comparisons are limited by differences in extraction solvent, assay format, and expression of results. Kaska [6] reported relatively high TPC and TFC values for an ethanolic *O. sipyleum* extract and observed activity in DPPH, ABTS, reducing-power, phosphomolybdenum, and metal-chelating assays. Zengin et al. [3] likewise demonstrated pronounced solvent-dependent differences in the antioxidant properties of *O. sipyleum* extracts and reported notable activity for the aqueous fraction. These literature findings suggest that *O. sipyleum* extracts exhibit antioxidant activity across different extraction systems, although the magnitude and characteristics of the measured responses remain dependent on extraction procedures and assay conditions.

The antioxidant behavior of the present *O. sipyleum* extract may be associated with its characteristic phenolic composition. Protocatechuic acid, the predominant targeted compound, is a 3,4-dihydroxybenzoic acid whose catechol-type structure contributes to its radical-scavenging and reducing properties. Previous in vitro studies have demonstrated the antioxidant activity of protocatechuic acid in DPPH, ABTS, and metal-reducing assays, with hydrogen-atom and electron-donation mechanisms proposed to contribute to these effects [22]. The high concentration of protocatechuic acid in the present extract may therefore contribute to its pronounced radical-scavenging and reducing capacities; however, such an association cannot establish a direct causal relationship.

Rosmarinic acid, caffeic acid, luteolin, and apigenin may also contribute to the antioxidant properties of the *O. sipyleum* extract. These compounds have been associated with the antioxidant activity of *Origanum* and other Lamiaceae extracts in previous phytochemical and bioactivity studies [13,23,24]. However, their individual contributions to the antioxidant responses observed in the present crude extract were not experimentally determined.

An apparently contrasting situation was observed for *S. pisidica*. Despite containing a markedly higher concentration of chlorogenic acid (3525.31 µg/g extract) than any individual targeted phenolic quantified in *O. sipyleum*, *S. pisidica* exhibited lower TPC, TFC, DPPH, ABTS, CUPRAC, and FRAP values. Chlorogenic acid has documented radical-scavenging and redox-related properties [25,26]. Therefore, the lower antioxidant responses of the *S. pisidica* extract should not be interpreted as evidence of weak intrinsic antioxidant activity of chlorogenic acid. The observed differences may reflect the broader chemical composition of the extracts, constituent interactions, and assay-specific reaction mechanisms [27,28]. Because the targeted LC–MS/MS analysis covered only 28 predefined phenolic compounds, the complete chemical composition underlying these responses remains uncharacterized.

This distinction is particularly important when considering TPC. The Folin–Ciocalteu assay is widely used to estimate total phenolic content; however, the reaction is based primarily on reducing capacity and is not completely specific to phenolic compounds. Non-phenolic reducing substances, including ascorbic acid and other redox-active constituents, may also contribute to the measured response [29,30]. Consequently, TPC values obtained by the Folin–Ciocalteu assay should not be expected to correspond directly to the summed concentrations of individual phenolic compounds quantified by targeted LC–MS/MS. The relatively high chlorogenic acid concentration but lower Folin–Ciocalteu response of *S. pisidica* therefore does not represent an analytical contradiction, because the two approaches measure fundamentally different analytical properties. In addition, spectrophotometric measurements of complex plant extracts may be affected by matrix-dependent background absorbance, overlapping spectral responses, and interactions among extract constituents. Furthermore, differences in assay chemistry, reaction conditions, and calibration standards restrict direct numerical comparisons across antioxidant tests [28]. Consequently, the results of the individual assays should be interpreted as complementary, method-dependent measurements rather than interchangeable estimates of a single antioxidant property.

Earlier work on *S. pisidica* also demonstrated substantial antioxidant activity but highlighted the strong influence of extraction solvent. Deveci et al. [9] reported that the methanol extract exhibited the highest antioxidant activity among the investigated *S. pisidica* extracts in most assays, whereas the hexane extract showed greater metal-chelating activity. Because that study employed organic solvents and expressed antioxidant activity using assay formats different from those used here, direct quantitative comparison with the present aqueous UAE extract is not appropriate. Nevertheless, both datasets support the conclusion that the antioxidant behavior of *S. pisidica* is strongly dependent on the chemical fraction recovered by the extraction procedure.

In contrast to the radical-scavenging and reducing-power assays, the phosphomolybdenum values of *O. sipyleum* (555.86 ± 50.90 mg TE/g extract) and *S. pisidica* (453.31 ± 8.37 mg TE/g extract) did not differ significantly. Similarly, ferrous-ion chelating activity was statistically comparable between the two species. These findings are consistent with the distinct chemical principles underlying the antioxidant assays. Metal chelation depends on the presence and accessibility of functional groups capable of coordinating Fe^2+^, and plant-derived chelators may include not only polyphenolic compounds but also other low- and high-molecular-weight ligands, such as amino acids, peptides, and polysaccharides [31,32]. Polyphenols containing catechol-type and related metal-binding sites are also well recognized for their ability to interact with iron ions [33]. Thus, the similar ferrous-ion chelating capacities of the two extracts despite their markedly different TPC, TFC, and targeted phenolic profiles further indicate that antioxidant behavior cannot be adequately characterized by a single chemical assay.

Finally, all antioxidant assays employed in this study are in vitro chemical assays. They provide comparative information on radical scavenging, reducing capacity, and metal chelation under controlled experimental conditions but do not establish antioxidant efficacy in vivo. Absorption, metabolism, biotransformation, tissue exposure, and interactions with endogenous antioxidant systems were not investigated and should therefore remain outside the interpretation of the present results.

### 3.3. Enzyme-Inhibitory Activities and Phytochemical Context

The two aqueous extracts exhibited distinct cholinesterase-inhibitory responses. *S. pisidica* showed significantly higher AChE inhibitor-equivalent activity than *O. sipyleum* (3.14 ± 0.08 versus 2.94 ± 0.09 mg GALAE/g extract; *p* < 0.05), whereas no statistically significant difference was observed in BChE inhibitor-equivalent activity between the two species (*p* > 0.05). Previous studies have also demonstrated cholinesterase-inhibitory activity in these species, although differences in extraction procedures and assay formats limit direct quantitative comparison. Zengin et al. [3] evaluated ethyl acetate, methanol, and water extracts of *O. sipyleum* and reported that the aqueous extract exhibited the highest AChE-inhibitory activity among the three extracts. Deveci et al. [9] reported AChE inhibition for *S. pisidica*, with the hexane extract producing 62.54 ± 0.88% inhibition under their experimental conditions [3,9]. Because these previous studies employed different solvents and reported activity using different analytical expressions, their values should not be directly compared with the GALAE-based results obtained in the present study.

The significantly higher tyrosinase-inhibitory activity observed for *S. pisidica*, despite its lower TPC and TFC, indicates that total phenolic or flavonoid abundance alone does not predict the inhibitory response. Previous studies have reported tyrosinase-inhibitory effects of p-coumaric acid, caffeic acid, rosmarinic acid, and ferulic acid [34,35]. Although some of these compounds were detected in the present extracts, their contributions to the observed interspecies difference were not directly investigated.

The two extracts showed statistically comparable α-amylase-inhibitory activities despite their different targeted phenolic profiles. Previous investigations have demonstrated that the effects of phenolic acids on α-amylase depend on molecular structure and assay conditions [36,37]. Song et al. [38] reported relatively weak α-amylase inhibition by chlorogenic acid compared with certain structurally related compounds.

A marked interspecies difference was observed for α-glucosidase inhibition. *O. sipyleum* exhibited an inhibitory activity of 319.56 ± 15.01 mg ACE/g extract, whereas no detectable inhibition was observed for *S. pisidica* within the investigated concentration range of 0.1–5.0 mg/mL. Previous studies have reported α-glucosidase-inhibitory effects of certain phenolic acids, including caffeic and protocatechuic acids [36], and demonstrated α-glucosidase inhibition by an *O. vulgare* extract containing rosmarinic acid [39].

The absence of detectable α-glucosidase inhibition in *S. pisidica*, despite the presence of chlorogenic and caffeic acids, does not establish or exclude the possible contributions of these individual constituents to enzyme inhibition. Likewise, the contrasting AChE responses and comparable BChE and α-amylase inhibitor-equivalent activities of the two extracts, despite substantial differences in their targeted phenolic profiles, demonstrate that the measured inhibitory responses are enzyme-dependent and cannot be predicted solely from the concentrations of individual phenolic compounds.

Finally, the enzyme-inhibition assays employed here represent in vitro screening endpoints and should not be interpreted as evidence of therapeutic efficacy. The biological effects of phenolic compounds in vivo depend strongly on their absorption, metabolism, circulating metabolites, and concentrations achieved in target tissues. Human bioavailability studies have shown substantial differences among polyphenols and have demonstrated that circulating metabolites may differ considerably from the parent compounds originally present in foods or plant extracts [40]. Consequently, the present results should be interpreted as comparative evidence of in vitro enzyme-inhibitory activity rather than as evidence of clinical or physiological effects.

### 3.4. Ethnobotanical Relevance and Study Limitations

The use of water as the extraction solvent was selected to maintain relevance to the traditional consumption of *Origanum* and *Sideritis* species as herbal teas, which are commonly prepared as aqueous infusions [41,42,43]. Water preferentially extracts relatively polar constituents, including many phenolic acids, flavonoid glycosides, and phenylethanoid glycosides, although the qualitative and quantitative composition recovered depends strongly on the plant matrix and extraction conditions [44]. The use of ultrasound-assisted extraction additionally enhances solvent penetration and mass transfer through acoustic cavitation and can improve the recovery of phenolic compounds compared with conventional extraction procedures [12,13].

Nevertheless, the UAE procedure employed in the present study should not be considered equivalent to household tea preparation. Traditional infusions typically involve steeping plant material in hot or near-boiling water for a limited period, whereas UAE introduces sonication and controlled extraction conditions that can enhance mass transfer and alter the recovery of individual constituents [12,13,41]. Accordingly, the concentrations and in vitro activities determined here represent standardized UAE–water extracts rather than direct estimates of compounds or activities obtained from routinely prepared herbal teas. Future studies directly comparing UAE extracts with standardized household-style infusions would help clarify the extent to which the present findings reflect actual consumer preparations. Previous studies have also indicated that prolonged ultrasonication and unfavorable extraction conditions may promote the oxidation or degradation of certain phenolic constituents, although these effects depend on the applied ultrasonic parameters and the chemical characteristics of the compounds [14]. Because extraction-time-dependent changes in individual phenolics were not investigated in the present study, the stability of the quantified compounds throughout the three-hour UAE procedure cannot be independently established.

Several aspects strengthen the present comparative design. Three independent aqueous extracts were prepared for each species, and all phytochemical and bioactivity analyses were performed independently on these extracts. Furthermore, both species were processed using the same extraction procedure and evaluated using the same analytical and bioactivity protocols, allowing direct comparison under standardized experimental conditions. The targeted LC–MS/MS method enabled quantitative determination of 28 predefined phenolic compounds, while the use of complementary antioxidant and enzyme-inhibition assays provided a broader assessment of in vitro bioactivity than reliance on a single analytical endpoint. An additional analytical limitation concerns the application of a previously validated LC–ESI–MS/MS method to plant matrices different from those used in its original validation. Although Cittan and Çelik [20] demonstrated acceptable analytical performance in olive leaf extracts, matrix-specific recovery and ionization effects were not independently evaluated for the aqueous extracts investigated here. The importance of matrix-specific evaluation is supported by Faccin et al. [45], who demonstrated that electrospray ion suppression and enhancement can differ substantially among medicinal plant extracts, including matrices with similar botanical origins. Accordingly, analytical performance established in one plant matrix cannot be assumed to be directly transferable to chemically distinct extracts without additional verification. Therefore, potential matrix-related influences on quantitative accuracy cannot be excluded, and the reported phenolic concentrations should be interpreted with this analytical limitation in mind.

Importantly, previous investigations have demonstrated variations in the phytochemical composition and biological activities of *O. sipyleum* and *S. pisidica* extracts obtained using different extraction solvents [3,9]. The present comparative findings extend this knowledge by demonstrating that markedly different targeted phenolic profiles are accompanied by distinct, assay-dependent bioactivity patterns under identical experimental conditions. In particular, the predominance of protocatechuic acid in the *O. sipyleum* extract and chlorogenic acid in the *S. pisidica* extract did not correspond to uniformly higher antioxidant or enzyme-inhibitory activities in either species. These findings indicate that the measured bioactivity of complex aqueous extracts cannot be inferred solely from the concentrations of their predominant targeted phenolic constituents. Nevertheless, these observations represent descriptive associations rather than evidence of direct mechanistic relationships.

The study also has several limitations. First, the targeted LC–MS/MS approach was restricted to 28 predefined phenolic compounds and therefore does not provide a comprehensive metabolomic characterization of either species. Other phenolic, non-phenolic, and potentially bioactive constituents may have contributed to the observed spectrophotometric and enzyme-inhibitory responses. Second, although three independent extracts were prepared from each species, the plant material originated from a single collection context; consequently, the present study does not account for possible intraspecific variation associated with genotype, geographical origin, altitude, environmental conditions, harvest time, or phenological stage, all of which can influence the phytochemical composition of medicinal and aromatic plants [46,47].

Third, the biological assays were conducted exclusively in vitro. Therefore, the observed antioxidant and enzyme-inhibitory activities should not be extrapolated directly to physiological or therapeutic effects. Polyphenols may undergo substantial transformation during digestion, absorption, phase II metabolism, and colonic microbial metabolism, and the metabolites reaching target tissues may differ considerably from the parent compounds present in the extracts [40]. Fourth, the present study does not establish causal relationships between individual phenolic compounds and specific bioactivities. The measured activities reflect the behavior of complex crude extracts and may result from combined effects among multiple constituents. Activity-guided fractionation, testing of isolated compounds, and reconstitution experiments would therefore be required to identify the constituents responsible for particular activities.

Finally, although α-glucosidase inhibition was clearly detected in *O. sipyleum*, no inhibition was detected for *S. pisidica* under the assay conditions. This finding represents a genuine difference between the two extracts rather than missing data; however, additional concentration-response experiments would be valuable to determine whether *S. pisidica* remains inactive across a broader concentration range. Taken together, the present findings should therefore be interpreted as a standardized comparative characterization of two UAE–water extracts rather than as direct evidence of the biological effects of traditionally consumed infusions.

## 4. Materials and Methods

### 4.1. Plant Materials and Botanical Identification

The aerial parts of *Sideritis pisidica* Boiss. et Heldr. and *Origanum sipyleum* L. were collected on 20 July 2025 from the Gemişpınarı locality, Çardak district, Denizli Province, Türkiye (37°46′37.47″ N, 29°52′04.84″ E), at an altitude of 900–1000 m above sea level. The *S. pisidica* specimen was collected by Ü. Ünlü and M. Dinç under collection number Ü. Ünlü 1001, whereas the *O. sipyleum* specimen was collected by Ü. Ünlü and M. Dinç under collection number Ü. Ünlü 1002. Taxonomic identification of both species was performed by Prof. Dr. Muhittin Dinç. Voucher specimens were deposited in the Herbarium of the Faculty of Science, Selçuk University (KNYA), Konya, Türkiye, where they are permanently preserved.

### 4.2. Preparation of Plant Material and Ultrasound-Assisted Aqueous Extraction

Following collection, the aerial parts of both species were dried in the shade under well-ventilated conditions for approximately 3–4 weeks until a constant mass was reached. The dried plant materials were subsequently reduced to small pieces using a laboratory blender, transferred to plastic containers, and stored at 4 °C until extraction.

For each species, three independent aqueous extracts were prepared (*n* = 3). For each independent extraction, 5 g of dried plant material was mixed with 100 mL of distilled water, corresponding to a solid-to-solvent ratio of 1:20 (*w*/*v*). The extraction mixture was placed in a capped 250 mL Erlenmeyer flask. Ultrasound-assisted extraction (UAE) was performed using an ultrasonic bath (DR-MH220, DZG SCIENCE, Konya, Türkiye) operating at a manufacturer-specified frequency of 40 kHz and rated ultrasonic power of 360 W for 3 h, following the general procedure described by Karakus [48]. The reported ultrasonic power represents the manufacturer’s specified equipment rating rather than the experimentally measured acoustic power delivered to the extraction medium. During sonication, the extraction vessels were externally cooled using an ice bath as required to maintain the ultrasonic bath water temperature at approximately 40 °C. The temperature was monitored using a digital thermometer immersed in the ultrasonic bath water rather than directly in the extraction mixture. Therefore, the reported temperature represents the surrounding bath water temperature, and the actual temperature inside the extraction vessel was not independently measured.

Following extraction, the mixtures were filtered through Whatman No. 1 filter paper to remove insoluble plant residues. The resulting aqueous extracts were frozen at −18 °C and subsequently lyophilized to dryness for approximately 48 h using a benchtop freeze dryer (FreeZone 2.5 L, Labconco Corporation, Kansas City, MO, USA) equipped with a −50 °C condenser. The dried extracts were transferred to airtight containers and stored at 4 °C until phytochemical and bioactivity analyses.

Extraction yield was calculated for each independently prepared extract as the percentage of dried extract obtained relative to the initial dry plant material. The extraction yield (%) was calculated using the following equation:

Extraction yield (%) = [Mass of lyophilized extract (g)/Initial mass of dried plant material (g)] × 100.

The extraction yield was calculated separately for each independent extraction and expressed as mean ± standard deviation (SD).

All subsequent LC–MS/MS, total phenolic and flavonoid, antioxidant, and enzyme-inhibitory analyses were performed independently on the three extracts prepared for each species.

The extraction conditions were selected based on a previously reported UAE procedure [48] and applied identically to both plant species to ensure methodological consistency. The solid-to-solvent ratio of 1:20 (*w*/*v*), extraction duration of 3 h, ultrasonic frequency of 40 kHz, and ultrasonic bath water temperature of approximately 40 °C were adopted as standardized operating conditions rather than established optimal parameters for either species. Previous research has demonstrated that ultrasound-assisted recovery of phenolic compounds is influenced by extraction parameters, including extraction time and temperature [12]. However, the present study was designed for comparative characterization rather than optimization of extraction conditions. Therefore, the selected extraction parameters should not be interpreted as optimal for either species. Establishing species-specific optimal extraction conditions would require a systematic investigation of extraction parameters and their effects on extraction yield, phenolic recovery, and the resulting phytochemical profiles.

### 4.3. Determination of Total Phenolic and Flavonoid Contents

Absorbance measurements were performed using a GENESYS 180 double-beam UV–Vis spectrophotometer (Thermo Fisher Scientific, Waltham, MA, USA).

The total phenolic content (TPC) of the extracts was determined using the Folin–Ciocalteu colorimetric method as previously described [49,50,51]. Briefly, 0.25 mL of the extract solution (2 mg/mL) was mixed with 1.0 mL of diluted Folin–Ciocalteu reagent (1:9, *v*/*v*) and mixed thoroughly. After 3 min, 0.75 mL of 1% Na_2_CO_3_ solution was added. No additional water was added to the reaction mixture, resulting in a final reaction volume of 2.0 mL. After incubation at room temperature for 2 h, the absorbance was measured at 760 nm. Gallic acid was used for calibration, and the results were expressed as mg gallic acid equivalents (GAE)/g extract. Because the Folin–Ciocalteu reagent can also react with non-phenolic reducing substances, the measured TPC values should be regarded as estimates of phenolic-related reducing capacity rather than specific measurements of total phenolic content. Accordingly, the reported TPC values represent gallic-acid-equivalent Folin–Ciocalteu responses and should not be interpreted as the absolute mass concentration of phenolic compounds in the extracts.

The total flavonoid content (TFC) was determined using the aluminum chloride colorimetric method as previously described [48,52]. Briefly, 1.0 mL of the extract solution (2 mg/mL) was mixed with an equal volume of 2% AlCl_3_ solution prepared in methanol. A corresponding sample blank was prepared by mixing 1.0 mL of the extract solution with 1.0 mL of methanol without AlCl_3_. No additional water was added to the reaction mixture, and the final reaction volume was 2.0 mL. After incubation at room temperature for 10 min, the absorbance was measured at 415 nm. The absorbance of the corresponding sample blank was subtracted from that of the sample. Rutin was used for calibration, and the results were expressed as mg rutin equivalents (RE)/g extract. Similarly, TFC values represent rutin-equivalent responses obtained using the aluminum chloride colorimetric assay and should not be interpreted as the absolute mass concentration of all flavonoid compounds present in the extracts.

Both TPC and TFC analyses were performed independently on three separately prepared extracts for each plant species (*n* = 3). The results were expressed as mean ± standard deviation (SD). The calibration curves for gallic acid and rutin were constructed over concentration ranges of 1–10 and 5–50 µg/mL, respectively. Both calibration curves demonstrated satisfactory linearity (R^2^ > 0.99). The corresponding regression equations and coefficients of determination are provided in Supplementary Table S3.

### 4.4. LC–MS/MS-Based Phytochemical Analysis

The targeted phenolic compositions of the ultrasound-assisted aqueous extracts of *O. sipyleum* and *S. pisidica* were analyzed using a previously validated liquid chromatography–electrospray ionization–tandem mass spectrometry (LC–ESI–MS/MS) method described by Cittan and Çelik [20]. A total of 28 phenolic compounds were targeted. Authentic reference standards were used for the identification of all 28 target analytes, as detailed in the Supplementary Materials. Prior to LC–MS/MS analysis, the lyophilized extracts were redissolved in ultrapure water at a concentration of 20 mg/mL. The resulting solutions were passed through a 0.45 µm membrane filter before chromatographic analysis. When necessary, the filtered solutions were further diluted with ultrapure water to ensure that the concentrations of the target analytes fell within their respective analytical calibration ranges. Analyses were performed using an Agilent 1260 Infinity liquid chromatography system (Agilent Technologies, Waldbronn, Germany) coupled to an Agilent 6420 Triple Quadrupole mass spectrometer equipped with an electrospray ionization (ESI) source.

Chromatographic separation was achieved on a Poroshell 120 EC-C18 column (100 × 4.6 mm, 2.7 μm; Agilent Technologies, Santa Clara, CA, USA) using water containing 0.1% (*v*/*v*) formic acid and methanol as the mobile phases. Phenolic compounds were identified by comparison of their retention times and multiple reaction monitoring (MRM) transitions with those of authentic reference standards and quantified using external standard calibration. Where necessary, sample solutions were appropriately diluted to ensure that analyte concentrations fell within their respective working calibration ranges (1–500 µg/L, depending on the analyte; Supplementary Table S2). The corresponding dilution factors were taken into account when calculating the final phenolic concentrations. Quantitative results were expressed as μg/g extract, referring to the mass of the dry, lyophilized extract. Analytical detection and quantification were distinguished using the identification criteria and sensitivity characteristics of the reference method. Analytes with identifiable signals below the applicable quantification limit were not considered reliably quantifiable, and such findings were distinguished from analytes for which no identifiable signal was observed. Because the reported LOD and LOQ values originated from the reference method validated in an olive leaf matrix, they were not regarded as independently established matrix-specific limits for the aqueous extracts investigated here.

Detailed chromatographic conditions, gradient elution program, LC–MS/MS operating parameters, MRM transitions, calibration equations, coefficients of determination (R^2^), limits of detection (LOD), and limits of quantification (LOQ) are provided in Supplementary Tables S1 and S2. The LC–ESI–MS/MS procedure was adapted from the previously validated analytical method developed by Cittan and Çelik [20]. The calibration equations, coefficients of determination (R^2^), analytical working ranges, and detection and quantification limits presented in Supplementary Table S2 were obtained from the original method validation study and were not independently re-established for the present plant matrices. The original validation also included assessments of intra-day and inter-day precision, analyte recovery, and matrix effects using olive leaf extracts. However, because the present study investigated aqueous extracts of *O. sipyleum* and *S. pisidica*, matrix-specific recovery, ion suppression/enhancement, and quantitative performance were not independently validated for these extracts. Consequently, the previously reported validation parameters should be regarded as characteristics of the reference analytical method rather than matrix-specific validation results for the samples investigated in this study. Because matrix-matched calibration, recovery, and ionization-effect experiments were not performed for the investigated aqueous extracts, the quantitative results should be interpreted as targeted estimates obtained through external-standard calibration, with potential matrix-dependent uncertainty.

### 4.5. Antioxidant Activity Assays

Absorbance measurements were performed using a GENESYS 180 double-beam UV–Vis spectrophotometer (Thermo Fisher Scientific, Waltham, MA, USA).

The antioxidant activities of the ultrasound-assisted aqueous extracts of *O. sipyleum* and *S. pisidica* were evaluated using six complementary in vitro assays representing different antioxidant mechanisms, including phosphomolybdenum total antioxidant capacity [53], DPPH radical scavenging [54], ABTS radical scavenging [55], cupric reducing antioxidant capacity (CUPRAC) [56], ferric reducing antioxidant power (FRAP) [57], and ferrous ion-chelating activity [58], with slight modifications [59,60,61,62,63].

All antioxidant assays were performed separately on the three independently prepared aqueous extracts of each species (*n* = 3). The experimental procedures, including reagent compositions, reaction volumes, incubation conditions, and absorbance measurement wavelengths, are described in the Supplementary Materials.

Trolox (6-hydroxy-2,5,7,8-tetramethylchroman-2-carboxylic acid) was used as the reference standard for the DPPH, ABTS, CUPRAC, FRAP, and phosphomolybdenum assays, whereas EDTA (ethylenediaminetetraacetic acid) was used as the reference standard for the ferrous ion-chelating assay. Antioxidant activities were expressed as mg Trolox equivalents (TE)/g extract or mg EDTA equivalents (EDTAE)/g extract, as appropriate. The reported values were calculated from the corresponding reference-standard calibration curves and normalized to the mass of the dry, lyophilized extract. The calibration concentration ranges were 10–50 µg/mL for the phosphomolybdenum assay, 0.5–10 µg/mL for the DPPH, ABTS, CUPRAC, and FRAP assays, and 1–10 µg/mL for the ferrous-ion chelating assay. All calibration curves demonstrated satisfactory linearity (R^2^ > 0.99). The corresponding regression equations and coefficients of determination are presented in Supplementary Table S4.

Antioxidant activities were reported as reference-standard equivalents to reflect the analytical endpoints of the six assays and to maintain consistency in the comparative evaluation of the two extracts. Although IC_50_ values can be determined for concentration-dependent radical-scavenging assays such as DPPH and ABTS, their calculation requires appropriate concentration–response data. The present antioxidant results were obtained and reported using Trolox- or EDTA-equivalent calibration approaches; therefore, IC_50_ values were not determined. Moreover, IC_50_ is not an appropriate primary endpoint for reducing-capacity assays such as CUPRAC and FRAP or for the phosphomolybdenum assay.

Additional details concerning sample preparation, experimental controls, and blank correction are provided in the Supplementary Materials.

### 4.6. Enzyme Inhibitory Activity Assays

Enzyme-inhibitory assays were performed using a Multiskan GO microplate spectrophotometer (Thermo Fisher Scientific Oy, Vantaa, Finland) with 96-well microplates.

The ultrasound-assisted aqueous extracts of *O. sipyleum* and *S. pisidica* were evaluated for inhibitory activity against acetylcholinesterase (AChE), butyrylcholinesterase (BChE), tyrosinase, α-amylase, and α-glucosidase using previously described spectrophotometric methods [48,64,65,66]. For the enzyme-inhibitory assays, stock solutions of the lyophilized extracts were prepared at a concentration of 20 mg/mL. Appropriate dilutions of these stock solutions were used to obtain the concentrations required for the individual assays. Acetylcholinesterase and butyrylcholinesterase inhibitory activities were determined according to Ellman-type procedures using galantamine as the reference inhibitor. Tyrosinase inhibitory activity was determined using kojic acid as the reference inhibitor, whereas acarbose was used as the reference inhibitor for the α-amylase and α-glucosidase assays.

The inhibitory activities were expressed as mg galantamine equivalents (GALAE)/g extract, mg kojic acid equivalents (KAE)/g extract, and mg acarbose equivalents (ACE)/g extract, as appropriate. These values represent enzyme-inhibitory activities expressed relative to the corresponding reference-inhibitor calibration standards and should not be interpreted as direct percentages of enzyme inhibition or as concentrations of individual bioactive constituents in the extracts. The calibration concentration ranges were 1–10 µg/mL for galantamine in the AChE and BChE assays, 10–100 µg/mL for kojic acid in the tyrosinase assay, and 0.5–5 µg/mL for acarbose in the α-amylase and α-glucosidase assays. Detailed assay protocols, reagent compositions, incubation conditions, and measurement parameters are provided in the Supplementary Materials. Sample blanks without the corresponding enzymes were used to correct for background absorbance, as described in the supplementary experimental procedures.

In addition to reference-inhibitor-equivalent activities, enzyme-inhibitory potency was evaluated by determining the half-maximal inhibitory concentration (IC_50_). Appropriate serial dilutions of the plant extracts and reference inhibitors were prepared to obtain concentration–response data. For the α-glucosidase assay, both extracts were tested over a concentration range of 0.1–5.0 mg/mL to evaluate concentration-dependent inhibitory activity. IC_50_ values were calculated by nonlinear regression analysis of the concentration-dependent percentage inhibition curves and expressed as mg/mL. The IC_50_ was defined as the concentration required to inhibit 50% of the corresponding enzyme activity. For α-amylase inhibition, neither plant extract achieved 50% inhibition at the highest tested concentration (20 mg/mL); therefore, the corresponding IC_50_ values were reported as >20 mg/mL without extrapolation beyond the experimentally investigated concentration range.

### 4.7. Statistical Analysis

For each species, three independent aqueous extracts were prepared (*n* = 3), and all phytochemical and bioactivity analyses were performed independently on these extracts. Where technical replicate measurements were performed for a given assay, the technical replicates were first averaged to obtain a single value for each independently prepared extract. Accordingly, the statistical unit throughout the study was the independent extract rather than the individual technical measurement. Results are expressed as the mean ± standard deviation (SD) of the three independent extracts. To account for differences in extraction yields, the targeted phenolic concentrations, total phenolic and flavonoid contents, and antioxidant activities were additionally expressed per gram of the initial dry plant material. For each independently prepared extract, normalization was performed individually by multiplying its extract-based measurement by its corresponding extraction yield expressed as a decimal fraction. The resulting three normalized values for each species were used to calculate the mean and standard deviation (SD). Statistical comparisons between the two species on a dry-plant-material basis were performed using Welch’s independent-samples *t*-test applied directly to these individually normalized values (*n* = 3 per species). Thus, neither group-mean extraction yields nor propagated summary statistics were used as the basis for the statistical comparisons.

Comparisons between *O. sipyleum* and *S. pisidica* for targeted phenolic concentrations, total phenolic and flavonoid contents, antioxidant activities, and reference-inhibitor-equivalent enzyme inhibitory activities were performed using Welch’s independent-samples *t*-test, which does not assume equal population variances. This approach was selected because variance homogeneity could not be reliably established with the limited number of independent extracts (*n* = 3 per species), and differences in variability were observed across several measured parameters. IC_50_ values were obtained by nonlinear regression analysis of concentration–response data. For IC_50_ values, differences among the two plant extracts and the corresponding reference inhibitor were assessed using one-way analysis of variance (ANOVA), followed by Tukey’s honestly significant difference (HSD) post hoc test. IC_50_ results reported as exceeding the highest tested concentration, including α-amylase IC_50_ values above 20 mg/mL, were excluded from ANOVA and post hoc comparisons. All statistical tests were two-sided. The significance threshold was set at *p* < 0.05. Given the exploratory nature of the study and the multiple phytochemical and bioactivity endpoints examined, the results of the individual comparisons were interpreted cautiously. No adjustment for multiple testing was applied, and the reported *p*-values should therefore be regarded as unadjusted, exploratory results. Given the small number of independent extracts (*n* = 3 per species) and the absence of a multiple-testing correction, statistically significant findings should be interpreted as exploratory evidence rather than confirmatory evidence of interspecies differences. Different superscript letters within the same parameter and reporting basis indicate statistically significant differences among the compared groups. Statistical comparisons were performed only when quantitative values were available for the groups being compared; compounds reported as not detected (ND) and enzyme activities for which no inhibition was detected (NI) were not included in the corresponding statistical comparisons. Measurements below an applicable limit of quantification were not treated as reliably quantifiable observations for inferential statistical comparisons. All statistical analyses were performed using IBM SPSS Statistics for Windows, Version 22.0 (IBM Corp., Armonk, NY, USA).

## 5. Conclusions

This study provides a standardized comparison of the targeted phenolic profiles and in vitro bioactivities of ultrasound-assisted aqueous extracts of *Origanum sipyleum* and *Sideritis pisidica*. The extracts exhibited distinct phenolic signatures, characterized by the predominance of protocatechuic acid in *O. sipyleum* and chlorogenic acid in *S. pisidica*. Importantly, the differences in phytochemical composition were accompanied by assay-dependent antioxidant and enzyme-inhibitory responses rather than a consistent superiority of either species across all biological endpoints.

These findings describe comparative associations rather than causal relationships between individual phenolic constituents and biological activities. Given the limited number of independent extracts (*n* = 3 per species) and the absence of correction for multiple comparisons, the statistical findings should be regarded as exploratory rather than confirmatory. The conclusions are restricted to the investigated plant materials and UAE–water extracts and should not be generalized to the species as a whole or to traditionally prepared herbal infusions. Future research should prioritize activity-guided fractionation to identify the constituents associated with the observed bioactivities.

## Figures and Tables

**Table 1:** Targeted LC–MS/MS phenolic profiles of *Origanum sipyleum* and *Sideritis pisidica* expressed per gram of extract and dry plant material.

No.	Compound	*O. sipyleum* (µg/g Extract)	*S. pisidica* (µg/g Extract)	*O. sipyleum* (µg/g Dry Plant)	*S. pisidica* (µg/g Dry Plant)
1	Gallic acid	ND	28.04 ± 0.75	ND	4.72 ± 0.42
2	Protocatechuic acid	2334.15 ± 24.20 ^a^	53.38 ± 1.47 ^b^	934.59 ± 50.88 ^a^	8.98 ± 0.79 ^b^
3	3,4-Dihydroxyphenylacetic acid	ND	ND	ND	ND
4	(+)-Catechin	1.08 ± 0.07 ^b^	2.40 ± 0.14 ^a^	0.43 ± 0.04 ^a^	0.40 ± 0.04 ^a^
5	Chlorogenic acid	4.06 ± 0.14 ^b^	3525.31 ± 15.77 ^a^	1.63 ± 0.10 ^b^	592.96 ± 49.78 ^a^
6	3-Hydroxybenzoic acid	252.54 ± 3.97 ^a^	90.43 ± 2.08 ^b^	101.12 ± 5.63 ^a^	15.21 ± 1.32 ^b^
7	4-Hydroxybenzoic acid	251.45 ± 1.48 ^a^	90.92 ± 1.26 ^b^	100.68 ± 5.41 ^a^	15.29 ± 1.30 ^b^
8	(−)-Epicatechin	ND	ND	ND	ND
9	Caffeic acid	129.31 ± 0.89 ^b^	320.19 ± 10.05 ^a^	51.78 ± 2.79 ^a^	53.86 ± 4.82 ^a^
10	Syringic acid	12.21 ± 1.01 ^b^	48.50 ± 1.27 ^a^	4.89 ± 0.48 ^b^	8.16 ± 0.72 ^a^
11	Vanillin	BLOQ	2.70 ± 0.24	BLOQ	0.45 ± 0.06
12	*p*-Coumaric acid	10.83 ± 0.99 ^b^	103.88 ± 1.88 ^a^	4.34 ± 0.46 ^b^	17.47 ± 1.50 ^a^
13	Taxifolin	ND	ND	ND	ND
14	Sinapic acid	ND	ND	ND	ND
15	Ferulic acid	2.30 ± 0.11 ^b^	196.75 ± 1.20 ^a^	0.92 ± 0.07 ^b^	33.09 ± 2.78 ^a^
16	Verbascoside	0.34 ± 0.03 ^b^	33.37 ± 0.73 ^a^	0.14 ± 0.01 ^b^	5.61 ± 0.49 ^a^
17	Luteolin 7-glucoside	0.18 ± 0.01 ^b^	2.30 ± 0.10 ^a^	0.07 ± 0.01 ^b^	0.39 ± 0.04 ^a^
18	Rosmarinic acid	122.23 ± 1.10 ^a^	8.60 ± 0.35 ^b^	48.94 ± 2.65 ^a^	1.45 ± 0.14 ^b^
19	Hyperoside	0.44 ± 0.02 ^b^	0.73 ± 0.01 ^a^	0.18 ± 0.01 ^a^	0.12 ± 0.01 ^b^
20	Hesperidin	ND	1.14 ± 0.07	ND	0.19 ± 0.02
21	Apigenin 7-glucoside	1.64 ± 0.06 ^b^	8.37 ± 0.40 ^a^	0.66 ± 0.04 ^b^	1.41 ± 0.14 ^a^
22	2-Hydroxycinnamic acid	ND	ND	ND	ND
23	Eriodictyol	ND	ND	ND	ND
24	Pinoresinol	ND	ND	ND	ND
25	Quercetin	ND	ND	ND	ND
26	Luteolin	39.37 ± 0.78 ^a^	3.71 ± 0.21 ^b^	15.76 ± 0.90 ^a^	0.62 ± 0.06 ^b^
27	Kaempferol	35.09 ± 2.86	ND	14.05 ± 1.37	ND
28	Apigenin	46.99 ± 0.65 ^a^	9.28 ± 0.50 ^b^	18.81 ± 1.04 ^a^	1.56 ± 0.16 ^b^

Values are expressed as mean ± standard deviation (SD). Phenolic compound concentrations are reported as µg/g extract and µg/g dry plant material. Dry-plant-based concentrations were calculated separately for each of the three independent extracts by multiplying its measured extract-based concentration by its corresponding extraction yield expressed as a decimal fraction. The resulting individually normalized values were used to calculate the mean and standard deviation (SD). Different superscript letters (a and b) indicate statistically significant differences between the two species (*p* < 0.05). Both extract-based and dry-plant-based comparisons were performed using Welch’s independent-samples *t*-test on the corresponding individual-extract measurements (*n* = 3 per species). BLOQ, below the limit of quantification, indicates that an identifiable analyte signal was observed but the calculated concentration was below the LOQ reported for the reference LC–MS/MS method. ND (not detected) indicates that no identifiable analyte signal meeting the applied identification criteria was observed under the analytical conditions. The LOD and LOQ values presented in Supplementary Table S2 were obtained from the previously published method validation [20] and were not independently established for the plant matrices investigated in the present study.

**Table 2:** Total phenolic and flavonoid contents of the ultrasound-assisted aqueous extracts of *Origanum sipyleum* and *Sideritis pisidica*, expressed per gram of extract and dry plant material.

Parameter	*O. sipyleum* (Per g Extract)	*S. pisidica* (Per g Extract)	*O. sipyleum* (Per g Dry Plant)	*S. pisidica* (Per g Dry Plant)
TPC (mg GAE)	118.88 ± 4.07 ^a^	47.88 ± 0.53 ^b^	47.60 ± 3.02 ^a^	8.05 ± 0.68 ^b^
TFC (mg RE)	32.67 ± 0.74 ^a^	13.81 ± 0.28 ^b^	13.08 ± 0.76 ^a^	2.32 ± 0.20 ^b^

TPC, total phenolic content; TFC, total flavonoid content; GAE, gallic acid equivalents; RE, rutin equivalents. TPC and TFC values are expressed as equivalents of the corresponding calibration standards and do not represent the absolute mass fractions of all phenolic compounds or flavonoids present in the extracts. Values are expressed as mean ± standard deviation (SD). Total phenolic and flavonoid contents are reported as mg GAE/g extract and mg RE/g extract, respectively, and as their corresponding equivalents per gram of dry plant material. Dry-plant-based values were calculated separately for each of the three independent extracts by multiplying its measured extract-based value by its corresponding extraction yield expressed as a decimal fraction. The resulting individually normalized values were used to calculate the mean and standard deviation (SD). Different superscript letters (a and b) within the same reporting basis indicate statistically significant differences between the two species (*p* < 0.05). Both extract-based and dry-plant-based comparisons were performed using Welch’s independent-samples *t*-test on the corresponding individual-extract measurements (*n* = 3 per species).

**Table 3:** Antioxidant activities of the ultrasound-assisted aqueous extracts of *Origanum sipyleum* and *Sideritis pisidica*, expressed per gram of extract and dry plant material.

Assay	*O. sipyleum* (Per g Extract)	*S. pisidica* (Per g Extract)	*O. sipyleum* (Per g Dry Plant)	*S. pisidica* (Per g Dry Plant)
Phosphomolybdenum (mg TE)	555.86 ± 50.90 ^a^	453.31 ± 8.37 ^a^	222.57 ± 23.62 ^a^	76.25 ± 6.55 ^b^
DPPH (mg TE)	286.62 ± 18.36 ^a^	90.53 ± 1.31 ^b^	114.76 ± 9.58 ^a^	15.23 ± 1.30 ^b^
ABTS (mg TE)	403.07 ± 19.88 ^a^	154.24 ± 12.84 ^b^	161.39 ± 11.74 ^a^	25.94 ± 3.07 ^b^
CUPRAC (mg TE)	499.44 ± 22.00 ^a^	189.76 ± 1.95 ^b^	199.98 ± 13.86 ^a^	31.92 ± 2.70 ^b^
FRAP (mg TE)	463.96 ± 10.53 ^a^	143.11 ± 4.73 ^b^	185.77 ± 10.79 ^a^	24.07 ± 2.17 ^b^
Ferrous-ion chelating (mg EDTAE)	13.05 ± 0.77 ^a^	14.38 ± 0.08 ^a^	5.23 ± 0.42 ^a^	2.42 ± 0.20 ^b^

TE, Trolox equivalents; EDTAE, ethylenediaminetetraacetic acid equivalents; DPPH, 2,2-diphenyl-1-picrylhydrazyl radical-scavenging activity; ABTS, 2,2′-azino-bis(3-ethylbenzothiazoline-6-sulfonic acid) radical-scavenging activity; CUPRAC, cupric reducing antioxidant capacity; FRAP, ferric reducing antioxidant power. Values are expressed as mean ± standard deviation (SD). Antioxidant activities are expressed as mg TE/g extract or mg EDTAE/g extract, as appropriate, and as the corresponding equivalents per gram of dry plant material. Because the six antioxidant assays are based on different chemical reactions and analytical conditions, their numerical values should not be directly compared across assays or interpreted as a single, unified measure of antioxidant capacity. Dry-plant-based values were calculated separately for each of the three independent extracts by multiplying its measured extract-based value by its corresponding extraction yield expressed as a decimal fraction. The resulting individually normalized values were used to calculate the mean and standard deviation (SD). Different superscript letters (a and b) within the same reporting basis indicate statistically significant differences between the two species (*p* < 0.05). Both extract-based and dry-plant-based comparisons were performed using Welch’s independent-samples *t*-test on the corresponding individual-extract measurements (*n* = 3 per species).

**Table 4:** Enzyme-inhibitory activities and IC_50_ values of the ultrasound-assisted aqueous extracts of *Origanum sipyleum* and *Sideritis pisidica*.

Enzyme Assay	*O. sipyleum*	*S. pisidica*	Standard
AChE (mg GALAE/g extract)	2.94 ± 0.09 ^a^	3.14 ± 0.08 ^b^	—
AChE IC_50_ (mg/mL)	1.11 ± 0.01 ^b^	1.05 ± 0.03 ^b^	0.0033 ± 0.0001 ^a^
BChE (mg GALAE/g extract)	5.15 ± 0.19 ^a^	5.08 ± 0.06 ^a^	—
BChE IC_50_ (mg/mL)	1.03 ± 0.01 ^b^	1.04 ± 0.02 ^b^	0.0054 ± 0.0004 ^a^
Tyrosinase (mg KAE/g extract)	21.01 ± 0.54 ^b^	23.90 ± 0.26 ^a^	—
Tyrosinase IC_50_ (mg/mL)	3.86 ± 0.10 ^c^	3.39 ± 0.04 ^b^	0.0809 ± 0.0003 ^a^
α-Amylase (mg ACE/g extract)	34.91 ± 1.11 ^a^	36.02 ± 1.07 ^a^	—
α-Amylase IC_50_ (mg/mL)	>20	>20	1.50 ± 0.01
α-Glucosidase (mg ACE/g extract)	319.56 ± 15.01	NI	—
α-Glucosidase IC_50_ (mg/mL)	1.75 ± 0.08 ^b^	ND	0.96 ± 0.01 ^a^

AChE, acetylcholinesterase; BChE, butyrylcholinesterase; GALAE, galantamine equivalents; KAE, kojic acid equivalents; ACE, acarbose equivalents; GALAE, KAE, and ACE values indicate reference-inhibitor-equivalent activities and do not represent direct inhibition percentages or the concentrations of individual inhibitory compounds. IC_50_, concentration required to achieve 50% enzyme inhibition; NI, no detectable inhibitory activity observed within the tested concentration range (0.1–5.0 mg/mL for α-glucosidase) under the applied assay conditions; ND, IC_50_ not determined because no measurable inhibition was observed within this range. The NI designation does not imply the absence of inhibitory potential under all experimental conditions. Equivalent inhibitory activities and IC_50_ values are expressed as mean ± standard deviation (SD) of three independent determinations (*n* = 3). Statistical comparisons of equivalent inhibitory activities between the two plant extracts were performed using Welch’s *t*-test, whereas IC_50_ values were analyzed using one-way analysis of variance (ANOVA) followed by Tukey’s honestly significant difference (HSD) post hoc test. Different superscript letters within the same row indicate statistically significant differences (*p* < 0.05). For α-amylase inhibition, the IC_50_ values of both plant extracts exceeded the highest tested concentration (20 mg/mL); therefore, no statistical comparison was performed for these values. Lower IC_50_ values indicate greater inhibitory potency.

## Data Availability

The original contributions presented in this study are included in the article/Supplementary Material. Further inquiries can be directed to the corresponding author.

## Supplementary Materials

The following supporting information can be downloaded at https://www.mdpi.com/article/10.3390/molecules31213956/s1. Table S1: LC–ESI–MS/MS parameters and analytical characteristics of the target phenolic compounds analyzed by multiple reaction monitoring in negative and positive ionization modes; Table S2: Calibration curves, linearity ranges, limits of detection, and limits of quantification of the LC–ESI–MS/MS reference method. Table S3: Calibration characteristics of total phenolic and flavonoid content assays; Table S4: Calibration characteristics of antioxidant activity assays.
