# Supplementary material for: Ultrasound-Assisted Aqueous Extraction of Origanum sipyleum and Sideritis pisidica: LC–MS/MS Phenolic Profiles and Bioactivities
Source: Molecules. 2026 Nov 2;31(21):3956. doi: 10.3390/molecules31213956 (PMC13765272; doi:10.3390/molecules31213956)
Supplement: Supplementary file 1 [file molecules-31-03956-s001.zip › molecules-4627067-supplementary.pdf]

Supplementary Materials

1. Chemicals

Gallic acid, (+)-catechin, chlorogenic acid, 4-hydroxybenzoic acid, (−)-epicatechin, caffeic acid, syringic acid, vanillin, taxifolin, sinapic acid, *p*-coumaric acid, ferulic acid, rosmarinic acid, 2-hydroxycinnamic acid, pinoresinol, quercetin, luteolin, and apigenin were purchased from Sigma-Aldrich (St. Louis, MO, USA). 3-Hydroxybenzoic acid, 3,4-dihydroxyphenylacetic acid, apigenin 7-glucoside, luteolin 7-glucoside, hesperidin, eriodictyol, and kaempferol were obtained from Fluka (St. Louis, MO, USA). Verbascoside, protocatechuic acid, and hyperoside were purchased from HWI Analytik (Ruelzheim, Germany). HPLC-grade methanol and formic acid were obtained from Sigma-Aldrich (St. Louis, MO, USA) and Merck (Darmstadt, Germany), respectively. Ultrapure water was produced using a Millipore Milli-Q Plus water purification system (Millipore Bedford Corp., Bedford, MA, USA).

2. Phytochemical Analysis

2.1. LC–ESI–MS/MS Analysis

The phenolic composition of the extracts was analyzed using a previously developed and validated liquid chromatography–electrospray ionization–tandem mass spectrometry (LC–ESI–MS/MS) method for the simultaneous determination of 28 phenolic compounds. Prior to chromatographic analysis, lyophilized plant extracts were redissolved in ultrapure water to obtain solutions at 20 mg/mL and filtered through 0.45 µm membrane filters. Where necessary, the filtered solutions were further diluted with ultrapure water to bring the target analyte concentrations within their respective calibration ranges. The dilution factors were accounted for when calculating the final concentrations, which were expressed as µg/g dry extract. Quantitative analyses were performed using an Agilent 1260 Infinity liquid chromatography system (Agilent Technologies) coupled to an Agilent 6420 Triple Quadrupole mass spectrometer equipped with an electrospray ionization (ESI) source.

Chromatographic separation was achieved on a Poroshell 120 EC-C18 column (100 mm × 4.6 mm I.D., 2.7 µm). The mobile-phase system, consisting of 0.1% formic acid and methanol, was selected because it provided satisfactory chromatographic resolution of the target analytes, including isomeric compounds, together with high analytical sensitivity. Solvent A consisted of 0.1% (*v*/*v*) formic acid in water, whereas solvent B was methanol. The gradient elution program was as follows: 0.00–3.00 min, 2% B; 6.00 min, 25% B; 10.00 min, 50% B; 14.00–17.00 min, 95% B; and 17.50 min, 2% B. The column temperature was maintained at 25 °C, the flow rate was 0.4 mL min^{-1}, and the injection volume was 2.0 µL.

The mass spectrometer was operated in both positive- and negative-ion multiple reaction monitoring (MRM) modes. The ESI source conditions were as follows: capillary voltage, −3.5 kV; gas temperature, 300 °C; gas flow rate, 11 L min^{-1}; and nebulizer pressure, 40 psi.

Phenolic compounds were identified by comparing their retention times and MRM transitions with those of authentic reference standards. Quantification was performed using external standard calibration. Detailed analytical parameters, including retention times, precursor ions, MRM transitions, calibration equations, coefficients of determination (R^2), limits of detection (LOD), and limits of quantification (LOQ), are presented in Supplementary Tables S1 and S2.

Supplementary Table S1. LC–ESI–MS/MS parameters and analytical characteristics of the target phenolic compounds analyzed by multiple reaction monitoring in negative and positive ionization modes.

Target compounds	Rt (min)	Precursor ion	MRM1 (CE, V)	MRM2 (CE, V)
Compounds analyzed by NI mode				
Gallic acid	8.891	168.9 [M − H]−	125.0 (10)	–
Protocatechuic acid	10.818	152.9 [M − H]−	108.9 (12)	–
3,4-Dihydroxyphenylacetic acid	11.224	167.0 [M − H]−	123.0 (2)	–
(+)-Catechin	11.369	289.0 [M − H]−	245.0 (6)	202.9 (12)
4-Hydroxybenzoic acid	12.439	136.9 [M − H]−	93.1 (14)	–
Caffeic acid	12.841	179.0 [M − H]−	135.0 (12)	–
Syringic acid	12.963	196.9 [M − H]−	181.9 (8)	152.8 (6)
3-Hydroxybenzoic acid	13.259	137.0 [M − H]−	93.0 (6)	–
Vanillin	13.397	151.0 [M − H]−	136.0 (10)	–
Verbascoside	13.589	623.0 [M − H]−	461.0 (26)	160.8 (36)
Taxifolin	13.909	303.0 [M − H]−	285.1 (2)	125.0 (14)
Sinapic acid	13.992	222.9 [M − H]−	207.9 (6)	163.8 (6)
p-Coumaric acid	14.022	162.9 [M − H]−	119.0 (12)	–
Ferulic acid	14.120	193.0 [M − H]−	177.8 (8)	134.0 (12)
Luteolin 7-glucoside	14.266	447.1 [M − H]−	285.0 (24)	–
Rosmarinic acid	14.600	359.0 [M − H]−	196.9 (10)	160.9 (10)
2-Hydroxycinnamic acid	15.031	162.9 [M − H]−	119.1 (10)	–
Pinoresinol	15.118	357.0 [M − H]−	151.0 (12)	135.7 (34)
Eriodictyol	15.247	287.0 [M − H]−	151.0 (4)	134.9 (22)
Quercetin	15.668	301.0 [M − H]−	178.6 (10)	151.0 (16)
Kaempferol	16.236	285.0 [M − H]−	242.8 (16)	229.1 (18)
Compounds analyzed by PI mode				
Chlorogenic acid	11.802	355.0 [M + H]+	163.0 (10)	–
(−)-Epicatechin	12.458	291.0 [M + H]+	139.1 (12)	122.9 (36)
Hesperidin	14.412	611.1 [M + H]+	449.2 (4)	303.0 (20)
Hyperoside	14.506	465.1 [M + H]+	303.1 (8)	–
Apigenin 7-glucoside	14.781	433.1 [M + H]+	271.0 (18)	–
Luteolin	15.923	287.0 [M + H]+	153.1 (34)	135.1 (36)
Apigenin	16.382	271.0 [M + H]+	153.0 (34)	119.1 (36)

R_t, retention time; NI, negative ion; and PI, positive ion. The chromatographic retention times, precursor ions, multiple reaction monitoring (MRM) transitions, and collision energies were adopted from the previously published LC–ESI–MS/MS method of Cittan and Çelik (2018). These parameters represent the reference analytical method and were not independently revalidated for the present plant matrices.

Supplementary Table S2. Calibration curves, linearity ranges, limits of detection, and limits of quantification of the LC–ESI–MS/MS reference method.

Compounds	Linearity and sensitivity characteristics				
	Range (µg/L)	Linear equation	R^2	LOD (µg/L)	LOQ (µg/L)
Gallic acid	5–500	y = 4.82x − 26.48	0.9988	1.46	4.88
Protocatechuic acid	2.5–500	y = 5.65x − 9.99	0.9990	1.17	3.88
3,4-Dihydroxyphenylacetic acid	5–500	y = 5.13x − 12.39	0.9990	1.35	4.51
(+)-Catechin	10–500	y = 1.45x + 1.95	0.9974	3.96	13.20
Chlorogenic acid	1–500	y = 12.14x + 32.34	0.9995	0.55	1.82
4-Hydroxybenzoic acid	5–500	y = 7.62x + 22.79	0.9996	1.72	5.72
(−)-Epicatechin	5–500	y = 9.11x − 9.99	0.9971	1.85	6.18
Caffeic acid	5–500	y = 11.09x + 16.73	0.9997	3.15	10.50
Syringic acid	10–500	y = 0.74x − 1.54	0.9975	3.75	12.50
3-Hydroxybenzoic acid	5–500	y = 3.69x − 12.29	0.9991	1.86	6.20
Vanillin	50–500	y = 2.02x + 135.49	0.9926	15.23	50.77
Verbascoside	2.5–500	y = 8.59x − 28.05	0.9988	0.82	2.75
Taxifolin	5–500	y = 12.32x + 9.98	0.9993	1.82	6.05
Sinapic acid	5–500	y = 2.09x − 6.79	0.9974	2.64	8.78
p-Coumaric acid	5–500	y = 17.51x + 53.73	0.9997	1.93	6.44
Ferulic acid	5–500	y = 3.32x − 4.30	0.9992	1.43	4.76
Luteolin 7-glucoside	1–500	y = 45.25x + 156.48	0.9996	0.45	1.51
Hesperidin	5–500	y = 5.98x + 0.42	0.9993	1.73	5.77
Hyperoside	2.5–500	y = 16.32x − 1.26	0.9998	0.99	3.31
Rosmarinic acid	1–500	y = 9.82x − 17.98	0.9989	0.57	1.89
Apigenin 7-glucoside	1–500	y = 21.33x − 31.69	0.9983	0.41	1.35
2-Hydroxycinnamic acid	1–500	y = 16.72x − 26.94	0.9996	0.61	2.03
Pinoresinol	10–500	y = 0.80x − 2.69	0.9966	3.94	13.12
Eriodictyol	2.5–500	y = 14.24x − 0.50	0.9998	0.80	2.68
Quercetin	5–500	y = 14.68x − 18.25	0.9997	1.23	4.10
Luteolin	5–500	y = 8.96x + 26.80	0.9992	1.34	4.46
Kaempferol	10–500	y = 0.82x − 3.06	0.9959	3.30	10.99
Apigenin	2.5–500	y = 11.29x + 38.05	0.9987	0.96	3.20

LOD and LOQ: limit of detection and limit of quantification, respectively. The calibration equations, coefficients of determination (R^2), analytical ranges, and limits of detection (LOD) and quantification (LOQ) were derived from the original validation study by Cittan and Çelik (2018), which was performed using an olive leaf matrix. These parameters were not independently determined or validated for the aqueous extracts of *O. sipyleum* and *S. pisidica*. Therefore, the reported values should not be interpreted as matrix-specific analytical performance characteristics of the present study.

2.2. Determination of Total Phenolic and Flavonoid Contents

Absorbance measurements for the total phenolic content (TPC) and total flavonoid content (TFC) assays were performed using a GENESYS 180 double-beam UV–Vis spectrophotometer (Thermo Fisher Scientific, Waltham, MA, USA).

The total phenolic content (TPC) was determined using the Folin–Ciocalteu method. Briefly, 0.25 mL of the sample solution was mixed with 1.0 mL of diluted Folin–Ciocalteu reagent (1:9, *v/v*) and mixed thoroughly. After 3 min, 0.75 mL of 1% Na_2CO_3 solution was added. The mixture was incubated at room temperature for 2 h, after which the absorbance was measured at 760 nm. A

reagent blank was prepared by replacing 0.25 mL of the sample solution with an equal volume of ultrapure water while maintaining the same Folin–Ciocalteu reagent (1.0 mL) and 1% Na_2CO_3 solution (0.75 mL) volumes. The blank was subjected to the same incubation conditions, and its absorbance was subtracted from the corresponding sample absorbance before quantification. Gallic acid was used as the calibration standard, and the results were expressed as mg gallic acid equivalents per gram of extract (mg GAE/g extract).

The total flavonoid content (TFC) was determined using the aluminum chloride colorimetric method. Briefly, 1.0 mL of the sample solution was mixed with 1.0 mL of 2% $AlCl_3$ solution in methanol. A corresponding blank was prepared by mixing 1.0 mL of the sample solution with 1.0 mL of methanol without $AlCl_3$. After incubation at room temperature for 10 min, the absorbance was measured at 415 nm. The absorbance of the blank was subtracted from that of the corresponding sample. Rutin was used as the calibration standard, and the results were expressed as mg rutin equivalents per gram of extract (mg RE/g extract).

Supplementary Table S3. Calibration Characteristics of Total Phenolic and Flavonoid Content Assays

Assay	Reference standard	Calibration range (μg/mL)	Regression equation	R^2
TPC	Gallic acid	1–10	y = 0.0778x + 0.0266	0.9956
TFC	Rutin	5–50	y = 0.0336x + 0.0044	0.9992

TPC, total phenolic content; TFC, total flavonoid content. In the regression equations, x represents the concentration of the corresponding reference standard (μg/mL), and y represents the measured absorbance. R^2 denotes the coefficient of determination obtained by linear regression analysis.

3. Antioxidant Activity

Absorbance measurements for all six antioxidant assays were performed using a GENESYS 180 double-beam UV–Vis spectrophotometer (Thermo Fisher Scientific, Waltham, MA, USA).

The total antioxidant capacity of the extracts was evaluated using the phosphomolybdenum assay. Briefly, 0.2 mL of the sample solution was mixed with 2.0 mL of reagent solution containing 0.6 M sulfuric acid, 28 mM sodium phosphate, and 4 mM ammonium molybdate. The reaction mixture was incubated at 95 °C for 90 min, after which the absorbance was measured at 695 nm.

The 1,1-diphenyl-2-picrylhydrazyl (DPPH) radical-scavenging activity was determined by mixing 1.0 mL of the sample solution with 4.0 mL of a 0.004% DPPH solution in methanol. The mixture was incubated at room temperature in the dark for 30 min, and the absorbance was subsequently measured at 517 nm.

For the ABTS radical-scavenging assay, the ABTS radical cation ($ABTS^{\bullet +}$) was generated by reacting 7 mM ABTS solution with 2.45 mM potassium persulfate and allowing the mixture to stand in the dark at room temperature for 12–16 h. Before use, the $ABTS^{\bullet +}$ solution was diluted with methanol to obtain an absorbance of 0.700 ± 0.02 at 734 nm. Subsequently, 1.0 mL of the sample solution was mixed with 2.0 mL of the diluted $ABTS^{\bullet +}$ solution. After incubation at room temperature for 7 min, the absorbance was measured at 734 nm.

Ferrous-ion chelating activity was determined by mixing 2.0 mL of the sample solution with 0.05 mL of 2 mM $FeCl_2$ solution. The reaction was initiated by the addition of 0.2 mL of 5 mM ferrozine solution. A corresponding blank was prepared by mixing 2.0 mL of the sample solution with 0.05

mL of 2 mM $FeCl_2$ solution and 0.2 mL of water without ferrozine. After incubation at room temperature for 10 min, the absorbance of the sample and blank was measured at 562 nm.

The cupric reducing antioxidant capacity (CUPRAC) was determined by mixing 0.5 mL of the sample solution with a reaction mixture containing 1.0 mL of 10 mM $CuCl_2$, 1.0 mL of 7.5 mM neocuproine, and 1.0 mL of 1 M NH_4Ac buffer (pH 7.0). A corresponding blank was prepared by mixing 0.5 mL of the sample solution with the reaction mixture without $CuCl_2$. After incubation at room temperature for 30 min, the absorbance of the sample and blank was measured at 450 nm.

Ferric reducing antioxidant power (FRAP) was determined using a freshly prepared FRAP reagent containing 0.3 M acetate buffer (pH 3.6), 10 mM 2,4,6-tris(2-pyridyl)-*s*-triazine (TPTZ) in 40 mM HCl, and 20 mM ferric chloride in a ratio of 10:1:1 (*v/v/v*). Briefly, 0.1 mL of the sample solution was mixed with 2.0 mL of FRAP reagent. Following incubation at room temperature for 30 min, the absorbance was measured at 593 nm.

Antioxidant activities were expressed as mg Trolox equivalents (TE)/g extract for the phosphomolybdenum, DPPH, ABTS, CUPRAC, and FRAP assays and as mg EDTA equivalents (EDTAE)/g extract for the ferrous-ion chelating assay. Trolox and EDTA were used as the respective reference standards. The calibration ranges, linear regression equations, and coefficients of determination (R^2) for the antioxidant assays are presented in Supplementary Table S4. All calibration curves demonstrated satisfactory linearity (R^2 > 0.99).

Supplementary Table S4. Calibration Characteristics of Antioxidant Activity Assays

Assay	Reference standard	Calibration range (μg/mL)	Regression equation	R^2
Phosphomolybdenum	Trolox	10–50	y = 0.0203x + 0.1708	0.9994
DPPH	Trolox	0.5–10	y = −0.0954x + 1.4351	0.9938
ABTS	Trolox	0.5–10	y = −0.1217x + 1.4943	0.9949
CUPRAC	Trolox	0.5–10	y = 0.0314x + 0.0440	0.9976
FRAP	Trolox	0.5–10	y = 0.0978x − 0.0207	0.9939
Ferrous-ion chelating	EDTA	1–10	y = −0.0826x + 1.6225	0.9937

DPPH, 2,2-diphenyl-1-picrylhydrazyl; ABTS, 2,2′-azino-bis(3-ethylbenzothiazoline-6-sulfonic acid); CUPRAC, cupric reducing antioxidant capacity; FRAP, ferric reducing antioxidant power; EDTA, ethylenediaminetetraacetic acid. In the regression equations, x represents the concentration of the corresponding reference standard (μg/mL), and y represents the measured absorbance. R^2 denotes the coefficient of determination obtained by linear regression analysis. The negative slopes observed for the DPPH, ABTS, and ferrous-ion chelating assays reflect concentration-dependent decreases in absorbance associated with radical scavenging or inhibition of Fe^{2+}–ferrozine complex formation.

4. Enzyme Inhibition Assays

Absorbance measurements for all enzyme-inhibitory assays were performed in 96-well microplates using a Multiskan GO microplate spectrophotometer (Thermo Fisher Scientific Oy, Vantaa, Finland).

The α-amylase-inhibitory activity was evaluated using the Caraway–Somogyi iodine/potassium iodide (IKI) method. Briefly, 25 μL of the sample solution was mixed with 50 μL of α-amylase solution (10 U/mL) prepared in 20 mM phosphate buffer (pH 6.9 containing 6 mM NaCl) in a 96-well microplate and pre-incubated at 37 °C for 10 min. The reaction was initiated by adding 50 μL of 0.05% starch solution and incubated for a further 10 min at 37 °C. A corresponding blank was

prepared using the same reaction mixture without α-amylase. The reaction was terminated by adding 25 μL of 1 M HCl, followed by 100 μL of iodine–potassium iodide solution. The absorbance of the sample and blank was measured at 630 nm, and the blank absorbance was subtracted from that of the corresponding sample.

For α-glucosidase-inhibitory activity, 50 μL of the sample solution was mixed with 50 μL of glutathione (2 mg/mL), 50 μL of α-glucosidase solution (0.2 U/mL) prepared in 0.1 M phosphate buffer (pH 6.8), and 50 μL of 10 mM p-nitrophenyl-α-D-glucopyranoside (PNPG) in a 96-well microplate. The reaction mixture was incubated at 37 °C for 15 min. A corresponding blank was prepared using the same reaction mixture without α-glucosidase. The reaction was terminated by the addition of 50 μL of 0.2 M sodium carbonate, and the absorbance of the sample and blank was measured at 400 nm. The blank absorbance was subtracted from that of the corresponding sample.

Tyrosinase-inhibitory activity was determined using a modified dopachrome method with L-DOPA as the substrate. Briefly, 25 μL of the sample solution was mixed with 40 μL of tyrosinase solution (200 U/mL) and 100 μL of phosphate buffer (pH 6.8) in a 96-well microplate and incubated at 25 °C for 15 min. The reaction was initiated by adding 40 μL of 10 mM L-DOPA. A corresponding blank was prepared using the same reaction mixture without tyrosinase. After incubation at 25 °C for 10 min, the absorbance of the sample and blank was measured at 492 nm.

Cholinesterase-inhibitory activity was evaluated using Ellman's method. Briefly, 50 μL of the sample solution was mixed with 125 μL of 0.3 mM DTNB and 25 μL of either acetylcholinesterase (AChE) (0.03 U/mL) or butyrylcholinesterase (BChE) (0.03 U/mL) solution in 50 mM Tris–HCl buffer (pH 8.0) in a 96-well microplate. Following incubation at 25 °C for 15 min, the reaction was initiated by adding 25 μL of 1.5 mM acetylthiocholine iodide (ATCI) or 1.5 mM butyrylthiocholine chloride (BTCl), respectively. A corresponding blank was prepared using the same reaction mixture without the enzyme solution. After incubation at 25 °C for 10 min, the absorbance of the sample and blank was measured at 405 nm. The blank absorbance was subtracted from that of the corresponding sample.

Enzyme-inhibitory activities were expressed as mg galantamine equivalents (GALAE)/g extract for AChE and BChE inhibition, mg kojic acid equivalents (KAE)/g extract for tyrosinase inhibition, and mg acarbose equivalents (ACE)/g extract for α-amylase and α-glucosidase inhibition. Galantamine, kojic acid, and acarbose were used as the respective reference standards. The calibration concentration ranges were 1–10 μg/mL for galantamine in the AChE and BChE assays, 10–100 μg/mL for kojic acid in the tyrosinase assay, and 0.5–5 μg/mL for acarbose in the α-amylase and α-glucosidase assays. For IC_{50} determination, serial dilutions of the plant extracts and corresponding reference inhibitors were prepared to establish concentration–response relationships. Percentage enzyme inhibition was determined at each tested concentration relative to the corresponding control, with correction for sample blanks. The resulting concentration–response data were fitted using nonlinear regression analysis, and IC_{50} values were calculated as the concentrations producing 50% inhibition of the respective enzyme activities. All IC_{50} values were expressed as mg/mL. When the maximum tested concentration did not produce 50% inhibition, the IC_{50} was reported as greater than the highest tested concentration rather than estimated by extrapolation.

References

Cittan, M., Çelik, A., 2018. Development and validation of an analytical methodology based on Liquid Chromatography–Electrospray Tandem Mass Spectrometry for the simultaneous determination of phenolic compounds in olive leaf extract. Journal of Chromatographic Science 56, 336–343.
